# Artificial Antigen-Presenting Cell Topology Dictates T Cell Activation

**DOI:** 10.1021/acsnano.2c06211

**Published:** 2022-08-15

**Authors:** Annelies C. Wauters, Jari F. Scheerstra, Irma G. Vermeijlen, Roel Hammink, Marjolein Schluck, Laura Woythe, Hanglong Wu, Lorenzo Albertazzi, Carl G. Figdor, Jurjen Tel, Loai K. E. A. Abdelmohsen, Jan C. M. van Hest

**Affiliations:** † Bio-Organic Chemistry, Institute for Complex Molecular Systems (ICMS), Eindhoven University of Technology, 5600 MB Eindhoven, The Netherlands; ‡ Department of Tumor Immunology, Radboud Institute for Molecular Life Sciences, Radboud University Medical Center, 6525 GA Nijmegen, The Netherlands; § Division of Immunotherapy, Oncode Institute, Radboud University Medical Center, 6525 GA Nijmegen, The Netherlands; ∥ Department of Biomedical Engineering, Institute of Complex Molecular Systems (ICMS), Eindhoven University of Technology, 5600 MB Eindhoven, The Netherlands; ⊥ Institute for Bioengineering of Catalonia (IBEC), The Barcelona Institute of Science and Technology (BIST), Barcelona 08036, Spain; # Institute for Chemical Immunology, 6525 GA Nijmegen, The Netherlands; ○ Laboratory of Immunoengineering, Department of Biomedical Engineering, Eindhoven University of Technology, 5600 MB Eindhoven, The Netherlands

**Keywords:** biodegradable polymersomes, artificial antigen-presenting cells, nano-immunotherapy, T cell activation, nanoparticle morphology, antibody density

## Abstract

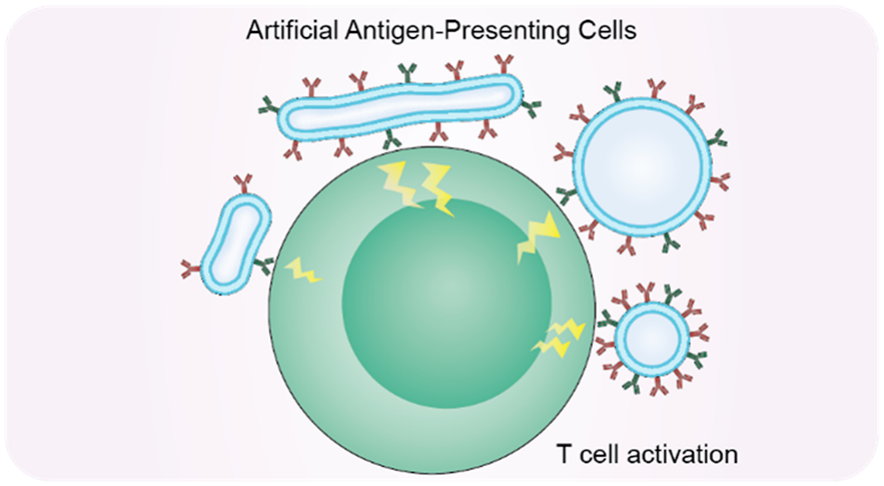

Nanosized artificial antigen-presenting cells (aAPCs), synthetic immune cell mimics that aim to activate T cells *ex* or *in vivo*, offer an effective alternative to cellular immunotherapies. However, comprehensive studies that delineate the effect of nano-aAPC topology, including nanoparticle morphology and ligand density, are lacking. Here, we systematically studied the topological effects of polymersome-based aAPCs on T cell activation. We employed an aAPC library created from biodegradable poly(ethylene glycol)-*block*-poly(d,l-lactide) (PEG-PDLLA) polymersomes with spherical or tubular shape and variable sizes, which were functionalized with αCD3 and αCD28 antibodies at controlled densities. Our results indicate that high ligand density leads to enhancement in T cell activation, which can be further augmented by employing polymersomes with larger size. At low ligand density, the effect of both polymersome shape and size was more pronounced, showing that large elongated polymersomes better activate T cells compared to their spherical or smaller counterparts. This study demonstrates the capacity of polymersomes as aAPCs and highlights the role of topology for their rational design.

## Introduction

Immunotherapy has revolutionized the treatment of diseases by activating or suppressing the patient’s own immune system.^1^ A range of different cell-based immunotherapies have been developed over the past years to generate effective antitumor T cell responses.^2^ Adoptive cell transfer (ACT) and dendritic cell (DC) vaccination exemplify such strategies and involve the isolation, *ex vivo* handling, and reinfusion of autologous immune cells to obtain or induce tumor-specific T cells.^3,4^ However, generation of effective cytotoxic T cell responses is hampered by low cell survival upon reinfusion, poor migration, and insufficient antigen presentation.^5^ Moreover, treatment protocols are patient-invasive, laborious, and costly.^6^ Nanomedicines have the potential to overcome these issues by generating off-the-shelf synthetic nanoparticles that can target and modulate specific immune organs and cells *in vivo*.^7−10^ For example, activatable nanomedicines have been shown to be effective in precise regulation of cancer immunotherapy.^11−13^ Particularly, artificial antigen-presenting cells (aAPCs), nano- or microparticles that replicate the function of natural APCs, have received much attention for their ability to activate T cells directly *in vivo*.^14−16^

aAPCs are designed to mimic the natural APC-T cell interface, known as the immunological synapse (IS). IS formation is initiated by binding of peptide-antigen presented on major histocompatibility complexes to T cell receptors (pMHC–TCR; signal 1), resulting in the activation of T cell signaling cascades and dynamic rearrangement of multiple interactive signals. To enhance the relatively low binding affinity of pMHC-TCR (i.e., 10^–4^ M), TCR/CD3 complexes (10–20 nm) preorganize in (linear) nanoclusters with average radii ranging from 35 to 70 to 300 nm in their longest dimension.^17,18^ Upon T cell activation, these nanoclusters localize with co-stimulatory molecules (e.g., with B7–CD28; signal 2) into larger microclusters, which subsequently organize in the central supramolecular activation cluster (cSMAC) surrounded by adhesion molecules (e.g., ICAM-1–LFA-1) in the peripheral supramolecular activation cluster (pSMAC); this leads to the formation of the so-called IS bull’s eye pattern (10–15 μm).^19,20^ In addition to signals 1 and 2, cytokine secretion (e.g., Interleukin(IL)-2 or IL-15; signal 3) is utilized to steer T cell activation. Sustained signaling correlates with the level of T cell activation that is manifested by upregulation of markers (e.g., CD69, CD25, or programmed cell death protein 1 (PD-1)), cytokine production (e.g., IL-2 and interferon (IFN)-γ), and T cell proliferation.^21−24^ aAPCs should thus be designed in a way that allows their optimal engagement with the TCR nano- or microclusters to effectively activate T cells.

Current strategies on designing aAPCs aim to mimic the signals displayed by natural APCs in a relatively simplified manner.^25^ Hereto, pMHC and co-stimulatory molecules are often replaced by anti-CD3 (αCD3) and anti-CD28 (αCD28) antibodies as signals 1 and 2, to stimulate T cells polyclonally. Commercially available αCD3/aCD28-coated magnetic microbeads (e.g., Dynabeads) are a classic example used for *ex vivo* T cell stimulation in ACT, although their large size renders them unsuitable for *in vivo* applications. Furthermore, these magnetic beads are rigid scaffolds, which do not provide the flexibility for dynamic signal arrangement nor the ability to encapsulate and release signals. In the last five decades, several other materials have been explored as aAPCs, such as liposomes, carbon nanotubes, iron oxide, and polymeric particles.^25−28^ Nanosized aAPCs constructed of biodegradable polymers have gained particular interest, due to their high physicochemical control and versatility and biocompatibility for *in vivo* applications.^29−31^ For example, the group of Fahmy developed aAPCs based on biodegradable poly(lactic-*co*-glycolic acid) (PLGA)-polymer micro- and nanoparticles functionalized with αCD3 or pMHC and αCD28, which provided sustained release of loaded IL-2 for *ex vivo* T cell stimulation.^32,33^

Recent studies have demonstrated that the topological parameters of nanosized aAPCs, including morphology (size and shape), functionality, and ligand density, determine optimal aAPC-T cell interaction and subsequent T cell activation.^34,35^ In this regard, elegant work has been performed by the group of Schneck, who demonstrated the beneficial effect of biodegradable PLGA-based aAPCs with an elongated shape on T cell activation.^36−39^ Moreover, they demonstrated the effect of both the particle size and the density of signaling molecules utilizing superparamagnetic iron oxide nanoparticles with different sizes and showed the importance of dynamic signal clustering through magnetic induction of smaller (50 nm) particles.^40−43^ Further studies showed that the density and co-localization of αCD3 and αCD28 or cytokines on a semiflexible polyisocyanopeptide (PIC) polymer scaffold were key in achieving an adequate T cell response.^44−46^ Although these investigations demonstrate the importance of aAPC topology on T cell activation, comprehensive studies that systematically evaluate the effect of multiple topological parameters of chemically equivalent nanosized aAPCs on T cell activation are lacking, mainly due to challenges in controlled engineering of such platform systems on the nanoscale.^9^

Previously, we developed biodegradable poly(ethylene glycol)-*block*-poly(d,l-lactide) (PEG-PDLLA) polymersomes and demonstrated their chemical and structural versatility.^47,48^ The flexibility of the employed PEG_22_-PDDLA_45_ polymer is influenced by the presence of organic solvent during self-assembly, which allows for control of both polymersome size as well as shape, through extrusion and osmotically induced shape transformation of spherical polymersomes into their tubular variants, respectively.^47,48^

**Figure 1:**
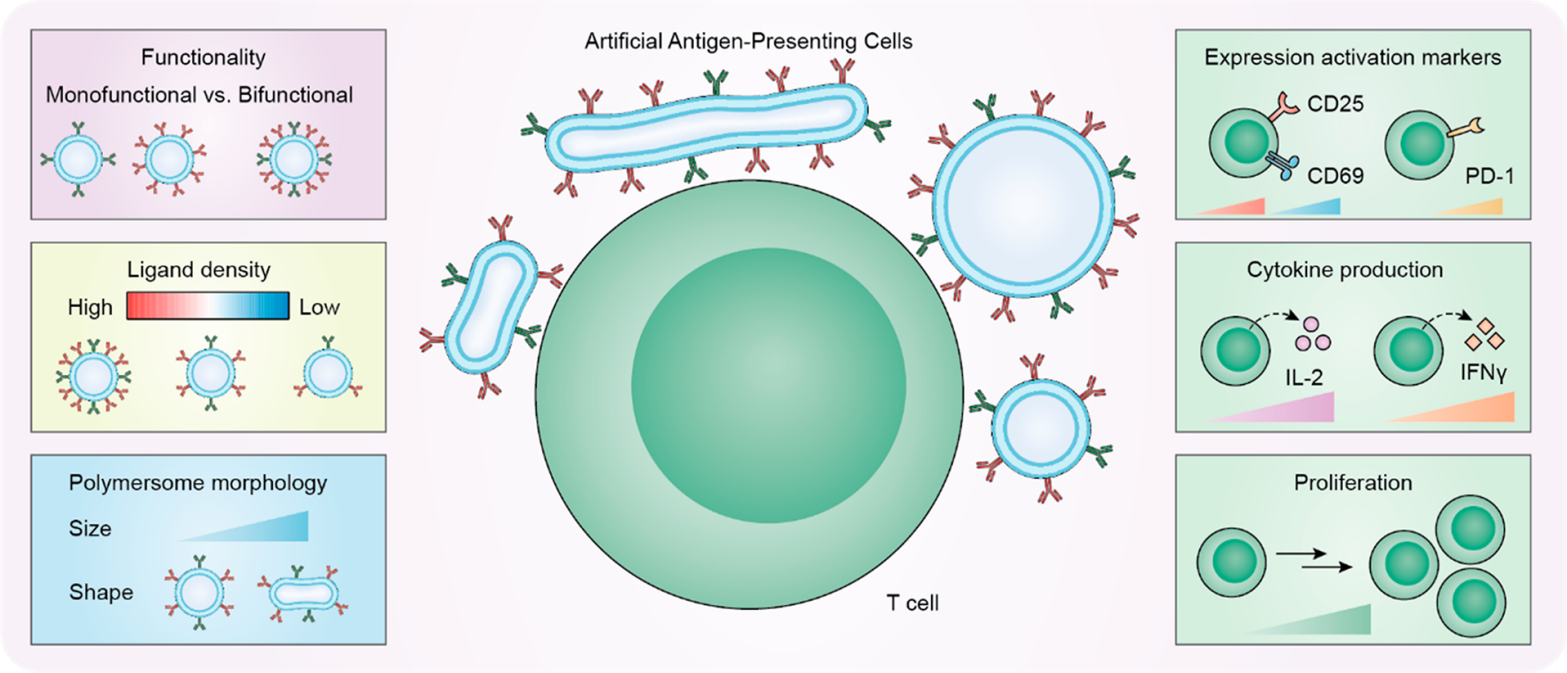
Schematic overview of the study design. We systematically studied the effect of polymersome-based artificial antigen-presenting cell topology, including functionality, ligand density, and morphology, on T cell activation. T cell activation was monitored through the expression of activation markers, production of cytokines, and proliferation.

Here, we have explored the potential of PEG-PDLLA polymersomes as an aAPC platform for T cell activation, by systematically examining the role of aAPC topology, including functionality, ligand density, and polymersome morphology. Hereto, we developed a library of aAPCs, comprising four morphologically different polymersomes, namely, small spheres (SmS), small tubes (SmT), large spheres (LgS), and large tubes (LgT), functionalized with either one or two antibodies (αCD3 and/or αCD28) with controlled densities. The effect of aAPC topology on T cell marker expression, cytokine production, and proliferation *ex vivo* was assessed (Figure 1). Due to the chemically identical composition of the platform, we could unambiguously demonstrate that larger size and higher antibody density were beneficial in T cell activation, whereas shape elongation was of importance at lower signal densities. This study therefore provides a rational design strategy for the further development of aAPC-based therapies.

## Results

### Engineering Polymersomes as Artificial Antigen-Presenting Cells

**Figure 2:**
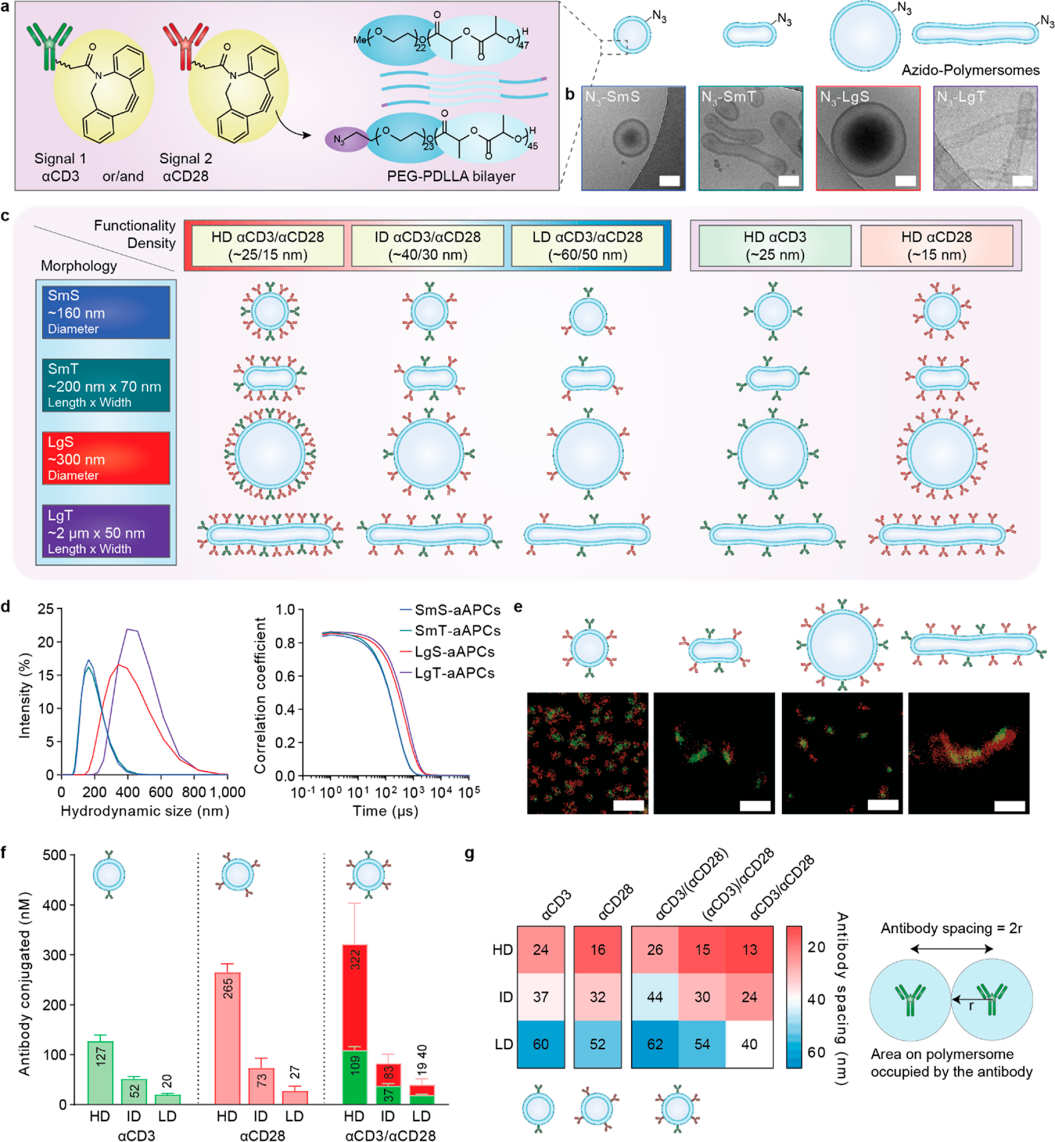
Development and characterization of a polymersome-based aAPC library with control over morphology and antibody density. (a) Conjugation of cyclooctyne-labeled and fluorescently labeled αCD3- and/or αCD28 antibodies to PEG-PDLLA azido-polymersomes with various morphologies. (b) Cryo-TEM images show the spherical or tubular morphology of the polymersomes. Scale bar = 100 nm. (c) Schematic overview of the aAPC library that was used for T cell activation experiments. (d) DLS intensity profiles and correlograms of polymersome-aAPCs. The data represents the mean (*n* = 15). (e) STORM imaging of bifunctional aAPCs indicates antibody conjugation on polymersome morphologies. Scale bar = 1 μm. (f) Mean antibody concentrations as determined by fluorescence spectroscopy and (g) mean antibody spacings for monofunctional αCD3- or αCD28-aAPCs and bifunctional αCD3/αCD28-aAPCs with different densities, as calculated from the mean antibody concentration and mean surface area concentration, determined from fluorescence spectroscopy and NTA, respectively. Average concentrations were calculated for four polymersome morphologies. The data represents the mean ± SD (*n* = 3 technical replicates, *N* = 4 polymersome morphologies). SmS = small spheres, SmT = small tubes, LgS = large spheres, LgT = large tubes, HD = high density, ID = intermediate density, LD = low density.

To systematically study the effect of aAPC features on T cell activation, we employed our well-defined biodegradable PEG-PDLLA polymersome platform with morphological control (Figure 2a).^47,48^ Four polymersome morphologies (small spheres (SmS), small tubes (SmT), large spheres (LgS), and large tubes (LgT)) were formed according to the solvent-switch methods previously described (Figure S1).^47^ Control over polymersome size was achieved by extrusion-based resizing of the particles after assembly. The tubular morphology was attained using an osmotically induced shape transformation process on both the originally formed polymersomes and the extruded ones. This polymersome formation methodology inherently implies that the four morphologies are chemically equivalent, as they are derived from one mother batch.

Analysis of the polymersomes using a combination of cryogenic transmission electron microscopy (cryo-TEM) imaging and dynamic light scattering (DLS) indicated a diameter of approximately 160 and 300 nm for SmS and LgS, respectively, whereas dimensions of SmT and LgT were approximately 200 nm × 70 nm and 2 μm × 50 nm (length × width) with aspect ratios of around 4 and 40, respectively (Figure 2b,c). Notably, these dimensions fall in the same size range of previously described nano- or microclusters for immune synapse formation. Additional data on polymersome characterization is shown in Figure S2.

In order to introduce functionality on the outer surface of the polymersomes, we assembled the polymersomes out of a mixture of nonfunctional block co-polymer (PEG_22_-PDLLA_47_, **3**) and azido-modified block co-polymer (N_3_-PEG_24_-PDLLA_45_, **5**) (5 wt %) (Figure S3). This allowed the conjugation of the required bioactive components via the well-established strain promoted azido-alkyne cycloaddition (SPAAC) click reaction (Figure 2a), at different densities.

We chose αCD3 (OKT3 clone) and αCD28 (9.3 clone) antibodies as model ligands, due to their well-known ability to trigger T cell activation by functioning as substitutes for signal 1 (pMHC-TCR; antigen presentation) and signal 2 (B7-CD28; co-stimulation) in the IS, respectively. Cyclooctyne-labeled and fluorescently labeled antibodies, αCD3-DBCO-ATTO488 and αCD28-DBCO-AF647, which allow for facile chemical conjugation and fluorescence quantification, were synthesized (Figure 2a and Table S1).

Next, polymersome-based aAPCs were attained by conjugating the aforementioned αCD3 or αCD28 antibodies, or their mixture, to the four azido-polymersome morphologies, yielding monofunctional αCD3- or αCD28-aAPCs and bifunctional αCD3/αCD28-aAPCs. Control over ligand density was achieved by adjusting the antibody/N_3_ molar ratio—by decreasing the initial feed of antibodies to lower the density. For bifunctional systems, αCD3/N_3_ and αCD28/N_3_ ratios were altered to sustain an αCD3/αCD28 1:2 ratio. Following the conjugation, the reaction was quenched and aAPCs were subsequently purified by centrifugation (Figure S4).

Finally, the obtained aAPCs were qualitatively and quantitively analyzed through a combination of nanoparticle characterization and fluorescence measurements, which allowed us to determine antibody spacings on the aAPCs using the following equation
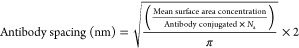
with the mean surface area concentration (∼3.4 nm^2^/mL) determined using nanoparticle tracking analysis (NTA), the conjugated antibody concentration (in nM) obtained by fluorescence spectroscopy analysis, and *N*_a_ being Avogadro’s number (6 × 10^23^ molecules/mol). With this approach, we created a library of 60 topologically different aAPCs. Analysis of the complete library can be found in the Supporting Information and indicated that the developed antibody conjugation methodology was highly controlled (Figures S4–S14).

DLS analysis of the aAPCs indicated that the integrity of the polymersomes was retained after antibody conjugation (Figure 2d). Furthermore, the stability of the aAPCs was confirmed by DLS measurements performed on aAPCs incubated in medium supplemented with human serum at 37 °C for 72 h (Figure S14).

Two-color stochastic optical reconstruction microscopy (STORM) imaging validated that both labeled antibodies (αCD3 and αCD28) were present on bifunctional aAPCs across the different morphologies, and the developed conjugation methodology did not compromise their spherical or tubular structure (Figure 2e and Figure S15).

A selection of the library was utilized for T cell activation studies; bifunctional aAPCs were attained at three different concentrations of both αCD3 and αCD28 (αCD3/αCD28 ratios approximately 1:1 to 1:4) as determined by fluorescence spectroscopy (Figure 2f), which generated αCD3 spacings of ∼25 (high density, HD), ∼40 (intermediate density, ID), or ∼60 nm (low density, LD) as calculated according to the above-described equation combining fluorescence spectroscopy and NTA measurements (Figure 2g). Monofunctional aAPCs with either αCD3 or αCD28 at high density were mixed in a 1:2 ratio, respectively, prior to T cell activation assays.

### Bifunctional aAPCs Enhance T Cell Activation Compared to Monofunctional aAPCs

**Figure 3:**
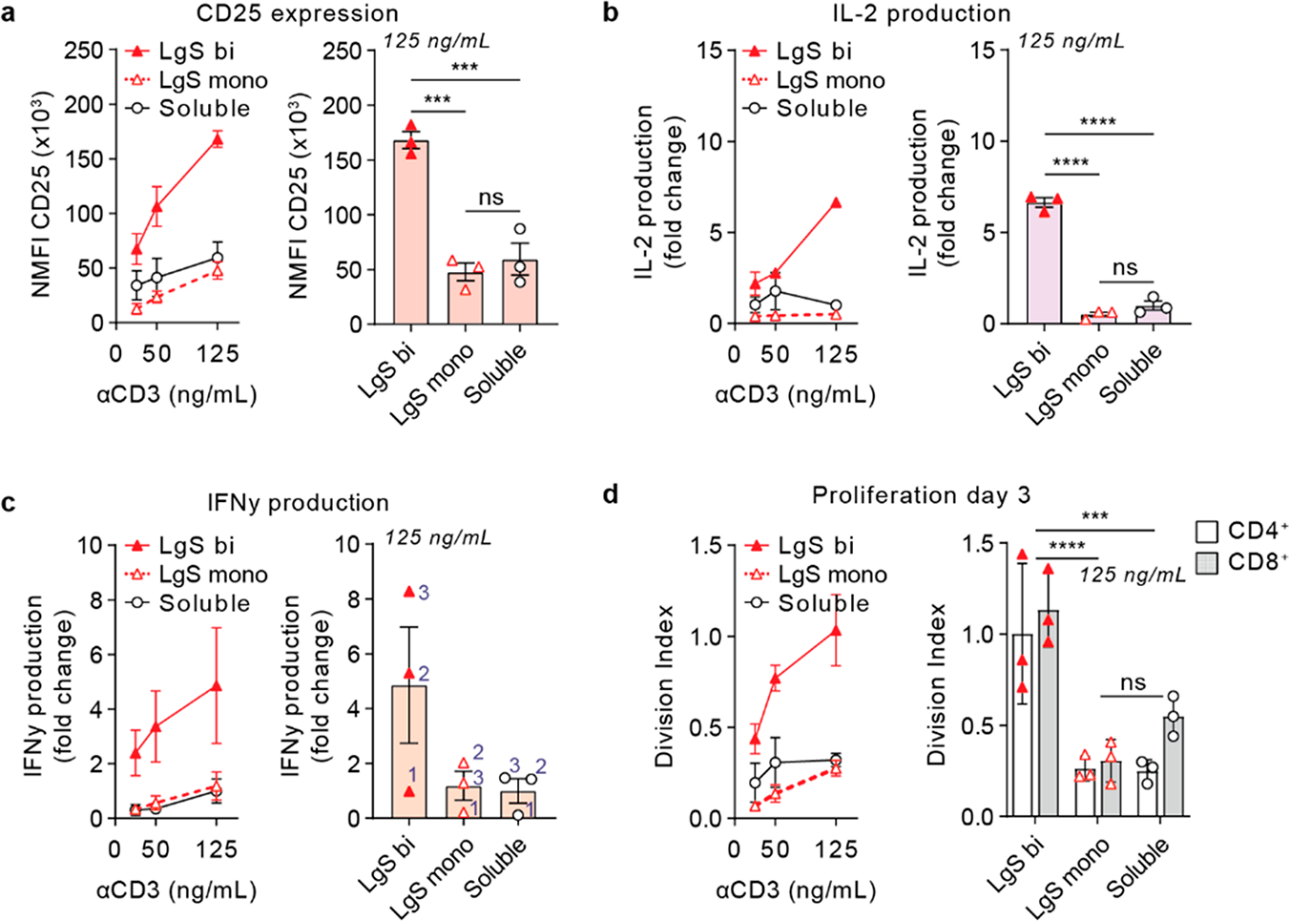
Bifunctional large spherical aAPCs enhance T cell activation compared to monofunctional aAPCs. T cells isolated from healthy donor buffy coats were stimulated with high density (HD) bifunctional (bi) or monofunctional (mono) large spheres (LgS) or soluble antibodies at αCD3 concentrations of 25, 50, and 125 ng/mL and αCD28 concentrations of ∼50, 100, and 250 ng/mL. (a) Normalized CD25 expression (freq × mean fluorescence intensity; NMFI), as determined with flow cytometry after 24 h. (b, c) Fold change in (b) interleukin (IL)-2 or (c) interferon (IFN)γ production, relative to soluble αCD3 + αCD28 at 125 ng/mL αCD3, as measured with ELISA after 24 h. (d) Division index (average number of cell divisions) of CD4^+^ and CD8^+^ T cells as determined through flow cytometric analysis of CellTrace Violet (CTV) fluorescence after 3 days of culture. All data is represented as the mean ± SE (*N* = 3 donors). Multiplicity adjusted *p*-values were calculated using a one-way or two-way analysis of variance (ANOVA) followed by Tukey’s multiple comparison test, ***p < 0.001, ****p < 0.0001.

Multivalent signaling (i.e., TCR signaling and co-stimulation) is essential for full T cell activation. To elucidate how preclustering of both signals affects T cell activation, primary human T cells (Figure S16) were stimulated with high ligand density bifunctional or monofunctional aAPCs or soluble antibodies at three different αCD3 concentrations of 25, 50, and 125 ng/mL and αCD28 concentrations of ∼50, 100, and 250 ng/mL. After 24 h, the expression of CD25 and production of cytokines (i.e., IL-2 and IFNγ) were measured for large spheres (Figure 3), as well as small spheres, small tubes, and large tubes (Figure S17), using flow cytometry (see Figure S16 for the gating strategy) and ELISA, respectively. T cell proliferation was assessed after 3 days of culturing through flow cytometric measurements using CellTrace Violet (CTV) fluorescence (Figure S16). Unfunctionalized polymersomes and Dynabeads were used as negative and positive controls, respectively (Figure S17). We compared the read-outs for monofunctional (mixture of CD3- and CD28-aAPCs) and bifunctional aAPCs and soluble antibodies to study the effect of functionalization on T cell activation.

Bifunctional large spheres upregulated CD25 to a higher degree than both monofunctional large spheres (*p* = 0.0005) and soluble antibodies (*p* = 0.0008; Figure 3a). Similarly, bifunctional large spheres increased the production of IL-2 in comparison with monofunctional counterparts and soluble antibodies (*p* < 0.0001; Figure 3b). IFNγ production also showed an increase following stimulation with bifunctional large spheres compared to monofunctional large spheres, which was evident for the individual donors, although not significant (Figure 3c). CTV analysis revealed that bifunctional large spheres caused T cells to proliferate more compared to monofunctional large spheres (*p* < 0.0001) or soluble antibodies (*p* = 0.0002; Figure 3d). We did not observe a difference between the proliferation of CD4^+^ and CD8^+^ T cell subsets.

Comparable to the results obtained for large spheres, bifunctional small spheres, small tubes, and large tubes enhanced CD25 expression and IL-2 and IFNγ production and proliferation, compared to their monofunctional counterparts (Figure S17). It was notable, however, that IFNγ production and three-day proliferation did not differ for monofunctional and bifunctional small spheres. This might be attributed to their small size and spherical shape, which allows the monofunctional small spheres to effectively cluster and sustain multivalent signaling comparable to bifunctional small spheres.

As a whole, these findings indicate that preclustering of αCD3 and αCD28 in close proximity (i.e., being co-displayed on the same polymersome) improves T cell activation. These results are in line with previous work on PIC polymer-based aAPCs.^44^

### High Ligand Density Enhances T Cell Activation in a Shape-Dependent Manner

**Figure 4:**
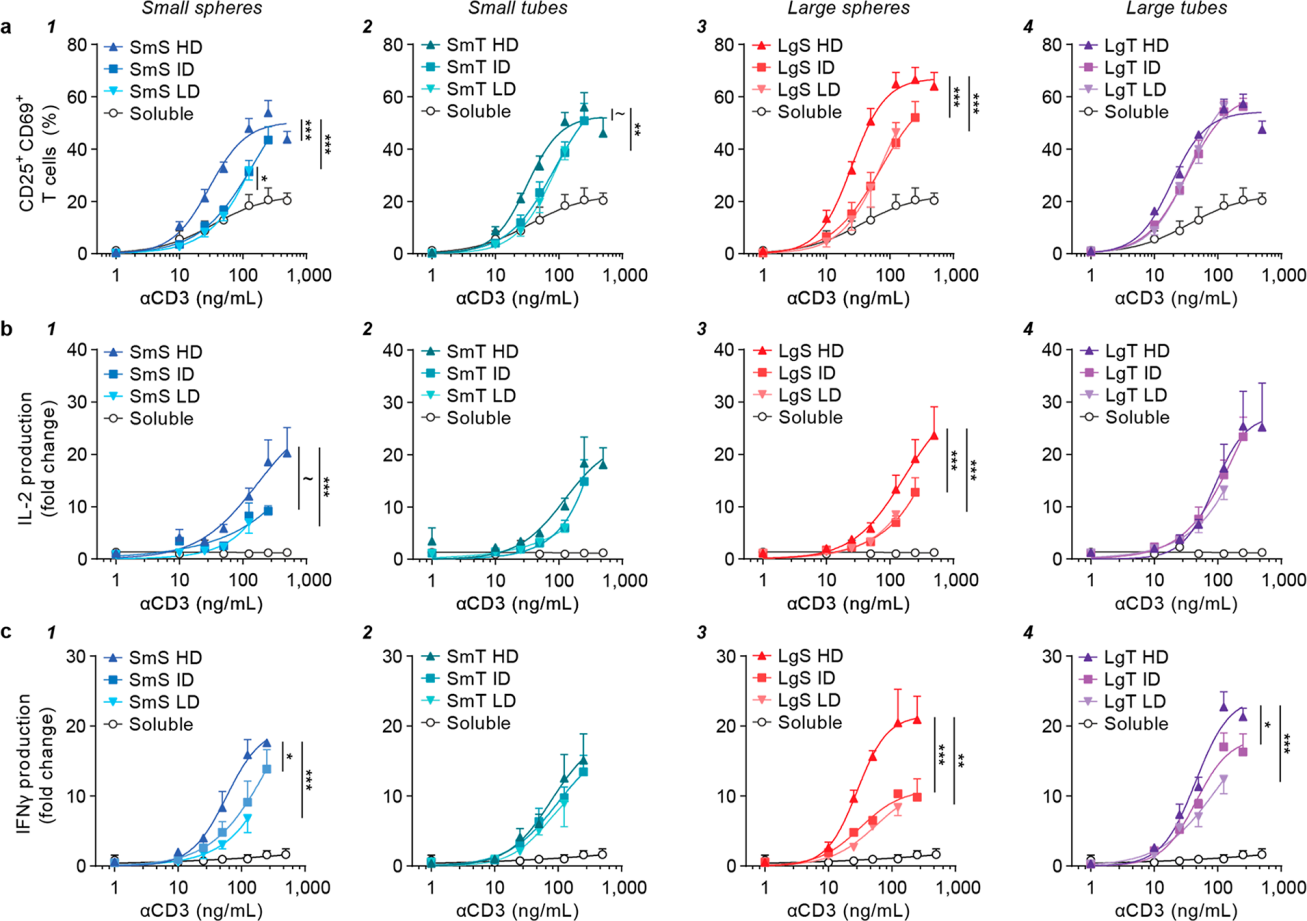
High ligand density enhances upregulation of early activation markers and cytokine production in a shape-dependent manner. T cells isolated from healthy donor buffy coats were stimulated with a library of high (HD), intermediate (ID), or low density (LD) bifunctional aAPCs or soluble antibodies at a range of αCD3 (1–500 ng/mL) and αCD28 (∼2–1000 ng/mL) concentrations. (a) CD25 and CD69 co-expression (freq) as determined with flow cytometry after 24 h. (b, c) Fold change in (b) IL-2 or (c) IFNγ production, relative to soluble αCD3 + αCD28 at 125 ng/mL αCD3, as measured with ELISA after 24 h. All data is represented as the mean ± SE (*N* = 3 donors, *n* = 2 replicates). Multiplicity adjusted *p*-values were calculated using an extra sum-of-squares F test followed by a Bonferroni–Dunn multiple comparison test, ∼*p* < 0.1, *p < 0.05, **p < 0.01, ***p < 0.001. All samples were significantly different compared to soluble antibodies with ***p < 0.001, unless otherwise indicated.

Sustained T cell signaling requires continual clustering of signaling molecules; providing TCR ligands in a closely spaced fashion promotes TCR microcluster formation.^17,24^ To determine whether αCD3/αCD28 density affects T cell activation, primary human T cells (Figure S16) were stimulated with bifunctional aAPCs from our library or soluble antibodies at 1–500 ng/mL αCD3 and ∼2–1000 ng/mL αCD28. The co-expression of the early activation markers CD25 and CD69 (percentage of CD25^+^/CD69^+^ T cells) as well as the production of IL-2 and IFNγ were quantified after 24 h (Figure 4 and see Figure S16 for the gating strategy). Moreover, aAPC binding was determined by quantifying the frequency of αCD3^+^ T cells after 6 h (Figure S18). To study the effect of ligand density, we compared the read-outs at high, intermediate, and low density for the different aAPC morphologies.

All aAPCs, regardless of ligand density or morphology, robustly enhanced CD25/CD69 expression and IL-2 and IFNγ levels relative to soluble antibodies (*p* < 0.013; Figure 4). Subsequently, we compared the effect of ligand density for small aAPCs. Small spheres showed enhanced CD25/CD69 expression levels as well as IL-2 and IFNγ production at high ligand density compared to intermediate and low density (CD25/CD69, *p* = 0.0006 for both; IL-2, *p* = 0.092 (ns) and *p* = 0.0006; IFNγ, *p* = 0.012 and *p* = 0.0003; Figure 4a–c/1). For small tubes, a high ligand density also significantly increased CD25/CD69 expression in comparison with low density (*p* = 0.0054), although this was not significant in comparison with intermediate density (*p* = 0.077; Figure 4a–c/2). Contrarily, ligand density did not significantly alter IL-2 or IFNγ production for small tubes.

Next, we investigated the effect of ligand density for the large aAPCs. In line with the findings for small spheres, large spheres showed the most pronounced enhancement in CD25/CD69 expression, IL-2, and particularly IFNγ production, at high ligand density compared to intermediate and low density (CD25/CD69, *p* = 0.0006 for both; IL-2, *p* = 0.0006 for both; IFNγ, *p* = 0.0003 and *p* = 0.003; Figure 4a–c/3). Intriguingly, large tubes showed no effect of ligand density on CD25/CD69 expression or IL-2 production (Figure 4a–c/4). However, large tubes showed enhanced IFNγ production at high ligand density, when compared to intermediate and low density (*p* = 0.018 and *p* = 0.0003, respectively), although this density effect was more pronounced for the large spheres.

The observed density effects on T cell activation were supported by aAPC binding analysis, which demonstrated that a higher ligand density increased the frequency of αCD3^+^ T cells (i.e., aAPC bound cells) for all morphologies (Figure S18). Furthermore, the observed dose–response curves on binding, as well as activation, indicate that plateaus are reached at ∼125 ng/mL αCD3 (notably at high density). This is in line with theoretical estimations on the added number of particles with respect to T cells, which suggests saturation of the cell surface at higher concentrations (Methods – T Cell Activation Assays).

Overall, these results indicate that for spherical aAPCs high ligand density enhances T cell activation, which is most pronounced for the large spheres. For tubular aAPCs, however, all ligand densities tested activate equally well.

### Large Tubular aAPC Morphology Improves T Cell Activation at Lower Ligand Densities

**Figure 5:**
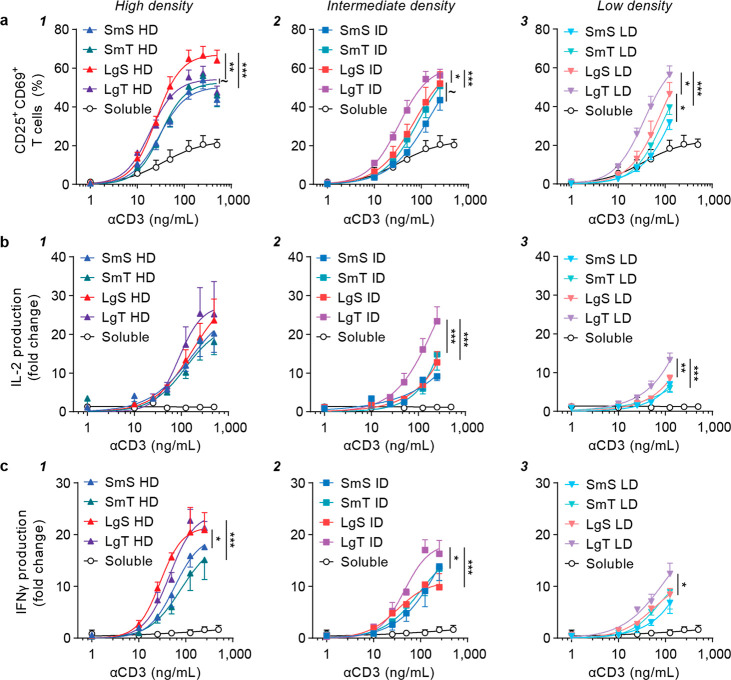
Large tubular aAPCs improve T cell activation at intermediate and low ligand density. T cells isolated from healthy donor buffy coats were stimulated with a library of bifunctional aAPCs with different polymersome morphologies or soluble antibodies at a range of αCD3 (1–500 ng/mL) and αCD28 (∼2–1000 ng/mL) concentrations. Notably, data is similar as in the previous figure but plotted differently to compare the effect of morphology. (a) CD25 and CD69 co-expression (freq) as determined with flow cytometry after 24 h. (b, c) Fold change in (b) IL-2 or (c) IFNγ production, relative to soluble αCD3 + αCD28 at 125 ng/mL αCD3, as measured with ELISA after 24 h. All data is represented as the mean ± SE (*N* = 3 donors, *n* = 2 replicates). Multiplicity adjusted *p*-values were calculated using an extra sum-of-squares F test followed by a Bonferroni–Dunn multiple comparison test, ∼*p* < 0.1, *p < 0.05, **p < 0.01, ***p < 0.001. All aAPCs were significantly different compared to soluble antibodies with ***p < 0.001, except for SmS LD with *p < 0.05.

To further elucidate the influence of aAPC size and shape on T cell activation, we compared the CD25/CD69 expression, as well as the IL-2 and IFNγ production and binding (αCD3^+^ T cells) for the different polymersome morphologies at high, intermediate, or low ligand density (Figure 5 and Figure S18).

First, we compared the effect of aAPC size on T cell activation. At high ligand density, large aAPCs increased CD25/CD69 expression compared to small aAPCs, which was most evident for the spheres (*p* = 0.0004) and only significant at trend level for the tubes (*p* = 0.057; Figure 5a/1). Large aAPCs also increased IL-2 production compared to small aAPCs at high ligand density, although this change was not significant (Figure 5b/1). Increasing the aAPC size at high ligand density resulted in elevated IFNγ levels, with large spheres and large tubes both outperforming small spheres (*p* = 0.013) and small tubes (*p* = 0.0004), respectively (Figure 5c/1). Findings on the effect of aAPC size at intermediate ligand density were comparable to those at low density. At these lower ligand densities, large aAPCs increased CD25/CD69 expression compared to small aAPCs, although it was most profound for the tubes (*p* = 0.0004 at both ID and LD) instead of the spheres, where only a trend could be observed at intermediate ligand density (*p* = 0.067), though the effect was significant at low density (*p* = 0.022; Figure 5a/2,3). Additionally, large tubes significantly enhanced IL-2 production compared to small tubes at intermediate and low ligand density (both *p* = 0.0004) and IFNγ production at intermediate density (*p* = 0.017), whereas no effect of size was observed for the spheres (Figure 5b,c/2,3).

We subsequently compared the spherical and tubular aAPCs to delineate the effect of shape on T cell activation. At high ligand density, large spheres showed higher CD25/CD69 expression than large tubes (*p* = 0.0052), although this shape effect was not observed for IL-2 and IFNγ production (Figure 5a–c/1). Importantly, however, at intermediate and low ligand density, large tubes significantly enhanced CD25/CD69 expression compared to large spheres (*p* = 0.028 at ID and *p* = 0.017 at LD; Figure 5a/2,3). This was also the case for IL-2 and IFNγ production at intermediate and low ligand density (IL-2, *p* = 0.0004 and *p* = 0.0016; IFNγ, *p* = 0.0008 and *p* = 0.039 for ID and LD, respectively; Figure 5b,c/2,3). In line with these findings for large aAPCs, similar trends on the effect of shape were observed for small aAPCs, although the effects were less pronounced and not significant. Small spheres enhanced IFNγ production compared to small tubes at high ligand density, whereas this shape effect was reversed at lower densities, where small tubes generally increased CD25/CD69 expression and cytokine production compared to small spheres.

Small spheres and small tubes showed approximately equal binding (Figure S18). Large spheres showed lower αCD3^+^ T cell frequencies than the other morphologies at high and intermediate ligand density, which might be attributed to their large spherical morphology that limits the effective amount of αCD3 that is available to bind. At all densities, the large tubular morphology enhanced the frequency of αCD3^+^ T cells compared to the other morphologies, indicating enhanced binding.

In general, these results demonstrate an effect of aAPC size and shape on T cell activation, which is dependent on the ligand density. A size effect was most clearly noticed for IFNγ secretion at high ligand density, where the larger spheres and tubes outperformed their smaller counterparts. At lower ligand densities, the effect of shape was more prominent, as large tubes more strongly upregulate CD25/CD69 expression and likewise induce the highest IL-2 and IFNγ production at intermediate and low density.

### aAPC Topology Affects PD-1 Expression and Proliferation after Three Days

**Figure 6:**
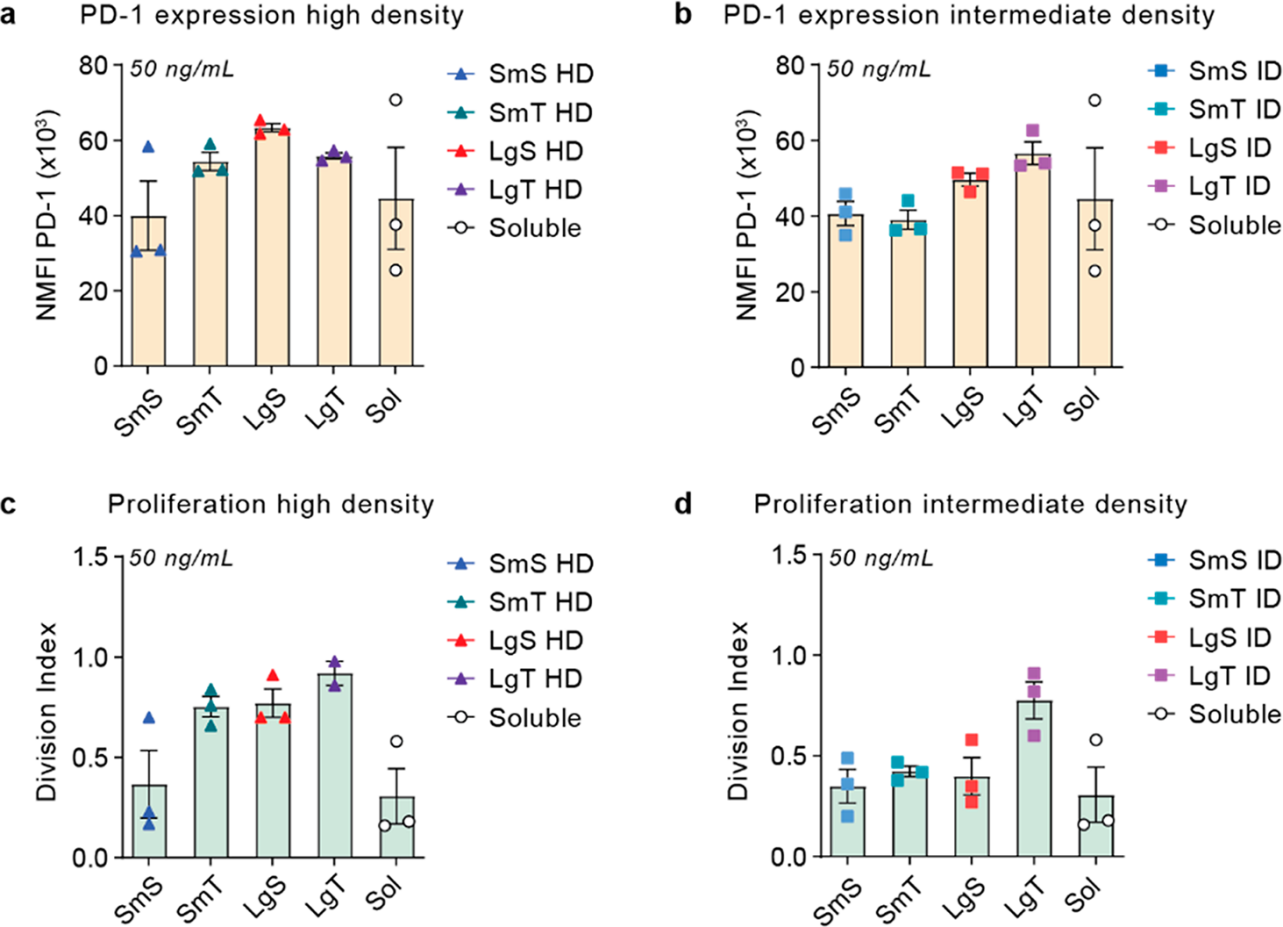
Large aAPC size and tubular shape enhance T cell activation and proliferation after 3 days. T cells isolated from healthy donor buffy coats were stimulated with a library of bifunctional aAPCs at high (HD) or intermediate (ID) ligand density with different polymersome morphologies or soluble antibodies at 50 ng/mL αCD3 and ∼100 ng/mL αCD28. (a, b) Normalized programmed cell death protein 1 (PD-1) expression (freq × mean fluorescent intensity; NMFI), as determined with flow cytometry after 3 days of culture for (a) HD or (b) ID aAPCs. (c, d) Division index of T cells as determined through flow cytometric analysis of CTV fluorescence after 3 days of culture for (c) HD or (d) ID aAPCs. All data is represented as the mean ± SE (*N* = 3 donors).

Immunotherapeutic efficacy of aAPCs depends on their ability to induce robust T cell activation and proliferation. To appraise the ability of our aAPCs with high and intermediate ligand density to induce a prolonged T cell response, the upregulation of PD-1, a T cell activation and exhaustion marker, and T cell proliferation were analyzed after 3 days of culturing with our aAPCs at 25, 50, and 125 ng/mL αCD3 and ∼50, 100, and 250 ng/mL αCD28 (Figure 6 and Figure S19; see also Figure S16 for the gating strategy).

First, we compared the effect of ligand density on PD-1 expression and proliferation after 3 days. In correlation with our previous findings, a high ligand density increased PD-1 expression for small spheres, small tubes, and large spheres compared to their intermediate density variants; for large tubes, only minor differences were observed (Figure S19). In the case of T cell proliferation, a marked ligand density effect was only observed for small tubes and large spheres.

Subsequently, we compared PD-1 expression and proliferation for different morphologies at high and intermediate ligand density. The effect of size and shape was analyzed at 50 ng/mL αCD3, which is below saturating concentrations and still in the linear range, based on our previous results (Figure 5 and Figure S18). At high ligand density, large spheres increased PD-1 expression more compared to small spheres, whereas there was no effect of size between the large and small tubes (Figure 6a). At intermediate ligand density, this effect of size was prevalent not only for spheres but also for tubes, as PD-1 expression was higher for large spheres compared to small spheres and large tubes compared to small tubes (Figure 6b). When investigating the effect of shape, small tubes showed higher PD-1 expression compared to small spheres at high ligand density, although expression levels were comparable at intermediate density. Large spheres mildly outperformed large tubes at high ligand density, whereas this shape effect was reversed at intermediate density.

Proliferation at high ligand density showed comparable effects of size and shape, although large tubes slightly enhanced the Division Index compared to large spheres (Figure 6c). Moreover, the size effect of large spheres was not recapitulated at intermediate ligand density, as large tubes showed increased proliferation compared to all other morphologies (Figure 6d).

Generally, high density affected three-day PD-1 expression and proliferation but not for small spheres and large tubes. Large size and tubular shape enhanced PD-1 expression and T cell proliferation at high density. At intermediate density, large size enhanced PD-1 expression, although proliferation appeared favored only by large tubes, which might be explained by the enhanced binding of LgT (Figure S18). These results corroborated our observations on T cell activation after 24 h, indicating that density and morphology have prolonged effects on T cell activation.

Notably, T cell activation, including CD25/CD69 expression, cytokine production, and PD-1 expression and proliferation at high and intermediate densities, reached similar levels as those resulting from control experiments using Dynabeads, indicating that our nanosized aAPCs, at the employed concentrations, have a comparable T cell activation capacity to the micron-sized beads (Figure S19 and Figure S20).

## Discussion

Nanoparticle-based aAPCs that mimic immune cell function have a high potential to replace cellular immunotherapies by activating T cells *in vivo*. Previous studies have demonstrated the importance of rational aAPC design, as nanoparticle topology affects nanoparticle–T cell interaction and T cell activation efficiency *in vitro* and *in vivo*. However, comprehensive studies that employ a chemically equivalent platform to systematically vary multiple topological parameters and delineate their impact on T cell activation are lacking, mainly due to difficulties in engineering of such platform systems.

In this study, we developed and employed a library of aAPCs with different topologies, based on chemically equivalent PEG-PDLLA polymersomes functionalized with αCD3 and αCD28 antibodies, to evaluate the role of ligand functionality, density, and nanoparticle morphology on T cell activation.

Previously, it has been demonstrated that co-display of αCD3 and αCD28 antibodies on the same entity enhances T cell activation.^44−46^ Our bifunctional aAPCs indeed enhanced CD25 expression, IL-2 and IFNγ production, and proliferation by T cells, compared to monofunctional aAPCs, demonstrating the advantage of preclustering both signals on one platform. It was notable however that monofunctional small spheres showed a similar effect on IFNγ production and T cell proliferation as their bifunctional counterparts, which can likely be explained by their small spherical morphology that supports dynamic clustering. This is in line with previous studies on magnetically clustered particles, which have also demonstrated that a mixture of monofunctional particles with similar sizes showed comparable T cell responses as bifunctional particles.^41^

Next, we studied the effect of ligand density on T cell activation. There was limited difference between the intermediate and low ligand densities; this can be explained by the fact that the 40 and 60 nm spacings lie within the reported size range of TCR/CD3 nanoclusters (35–70 nm),^17^ which therefore might prohibit that at these densities aAPCs can address multiple TCR/CD3 complexes within one cluster. A density that is below this threshold, for example, in line with the TCR/CD3 complex size of ∼10 nm,^49^ might be optimal, but this is difficult to achieve with large antibodies and would require, for example, the use of smaller natural ligands or bispecific antibodies.

High ligand density enhanced T cell activation compared to lower densities for spheres, which was most pronounced for large spheres, whereas for the small and particularly large tubes all densities activated T cells equally. This might be explained by the more rigid morphology of the large spheres, which requires a high ligand density to provide sufficient antibody numbers for effective T cell interaction. Although our polymer membranes are amorphous at room temperature (polymers have a glass transition temperature (*T*_g_) of ∼25 °C), we can assume limited fluidity compared to, for example, liposomes and restricted dynamics of the membrane-bound ligands. This can explain the importance of a high ligand density for large spheres. For tubular polymersomes, however, their worm-like structure can support dynamic clustering of signals at lower densities, by covering a larger cell surface area upon interaction, in line with previous studies.^50^ Future studies should focus on delineating the aAPC–T cell dynamic interaction (artificial immunological synapse) in greater detail and exploiting the use of responsive polymersomes that can adapt their topology for T cell interaction.

We further investigated the effect of the size and shape of different aAPC polymersomes on T cell activation. Notably, the dimensions of our polymersomes were in line with the reported dimensions of the TCR nanoclusters (35–70 nm up to 300 nm in the longest dimension) and microclusters (1–1.5 μm).^17−20^ We found that large spheres (∼300 nm) and large tubes (∼2 μm × 50 nm) enhanced T cell activation more compared to small spheres (∼160 nm) and small tubes (∼200 nm × 70 nm) at high ligand density. These results can be attributed to the increased surface area of larger particles, providing the necessary space for displaying a higher number of antibodies required for interaction with the nano- and microclusters on the T cell, in line with previous studies that compared the effect of nanoparticle size.^51^ Besides this effect of size, a tubular shape enhanced T cell activation, which was most prominent for the large tubes at lower ligand densities. Their more extended surface area can likely enhance the number of contact points between the aAPC and the T cell, allowing more antibodies to bind. The observed size and shape effects were also corroborated by the enhanced binding of large tubes to T cells compared to other morphologies. Finally, findings on topological effects were comparable when studying PD-1 expression and T cell proliferation after 3 days.

Several conclusions can be drawn from our studies, the most apparent being that (i) a semiflexible/tubular shape can enhance the potency and reduce the required concentration or ligand density and (ii) particles with larger size outperform their smaller counterparts. It however remains crucial to study how aAPCs’ topology affects their distribution *in vivo*. Together, these results provide potential guidelines for future development of aAPCs, where we primarily suggest the (rational) optimization of ligand density, as this clearly was the main determinant for effective activation. It is also important to note that extensive characterization, of both the nanoparticles and protein conjugation, is key for reliable comparative studies.

The current study was performed *ex vivo*, which is a passive environment, and topological effects might be different in the dynamic *in vivo* environment. We observed that the concentration of aAPCs was also one of the leading factors affecting T cell activation, and accordingly, an *in vivo* biodistribution supporting a local high aAPC concentration might be of primary importance to achieve high activation efficacy. Systematic studies that explore the effect of the topology of functionalized polymersomes on the immune organs and their therapeutic effect are thus crucial for optimal development.

The next step forward to improve the performance of our aAPC system, and also make it more suitable for *in vivo* applications, would be the introduction of antigen-specific ligands (pMHC). As an initial proof-of-concept, we suggest the use of a well-established antigen-specific model system. For example, aAPCs functionalized with MHC complexed with the OVA-peptide can be used to assess specific activation of OT-1 T cells both *in vitro* and *in vivo*. As a follow-up, such systems can be expanded to other (tumor-specific) antigens, such as NY-ESO-I or neo-antigens. We expect that differences due to topological effects will be more prevalent, due to the lower avidity of the peptide–MHC complex for the T cell receptor, which will further enhance the importance of ligand density and morphology.

Furthermore, other co-stimulatory signals (e.g., CD70, CD80/CD86, 4-1BBL, or OX40L) can be incorporated and the third signal in immune regulation, namely, the controlled release of cytokines (e.g., IL-2 or IL-12), can be included by encapsulation in the polymersome core.^14^

## Conclusions

In summary, we have demonstrated the potential of our biodegradable PEG-PDLLA polymersomes as an aAPC platform and systematically studied the effect of their topology, including functionality, ligand density, and morphology, on T cell activation. Co-display of αCD3 and αCD28 antibodies as well as a high ligand density and large size significantly enhanced T cell activation. aAPCs with a large tubular shape significantly enhanced T cell activation at lower densities compared to smaller and spherical aAPCs. These results highlight the importance of nanoparticle topology for future design of immunotherapeutic aAPCs.

## Methods

### Preparation of Azido-Functionalized PEG-PDLLA Large Spherical and Tubular Polymersomes (N_3_-LgS and N_3_-LgT)

In a 15 mL glass vial, PEG_22_-PDLLA_47_**3** (95 wt %) and N_3_-PEG_24_-PDLLA_45_**5** (5 wt %) were weighed and dissolved in a mixture of organic solvents (dioxane/THF, 4:1 v/v) to obtain a block co-polymer concentration of 10 mg/mL. The vial was capped with a lid, and the polymer solution was left to stir for approximately 30 min to ensure complete polymer dissolution. Thereafter, the polymer solution was transferred to a laminar flow cabinet to execute the following steps under sterile conditions. The polymer solution was filtered using a 0.2 μm PFTE filter (which is compatible with organic solvents) to remove impurities. The filtered polymer solution was added to a clean 15 mL glass vial (2 mL per vial), and a magnetic stirrer bar was added to the solution. Subsequently, the vial was capped with a rubber septum and the solution was stirred for approximately 5 min. Using a syringe pump, 2 mL (50 vol %) of endotoxin-free water was added at a rate of 1 mL/h. The obtained cloudy polymersome solution was directly transferred to a prehydrated dialysis membrane, and dialysis was performed at 4 °C—against precooled Milli-Q water (1 L)—for 24 h, with a water change after the first hour. For the formation of nanotubes, the polymersome solution was dialyzed against a 50 mM NaCl solution. Finally, the polymersome and nanotube solutions (Milli-Q and 50 mM NaCl solution, respectively) were replaced by PBS by dialyzing them against PBS (1 L), with a solution change (1 L) after 4 h. After another 4 h, the PBS dialysis solution was replaced by endotoxin-free PBS (2 L) for overnight dialysis. The resulting polymersome solutions in endotoxin-free PBS were taken from the dialysis bags and stored in endotoxin-free Falcon tubes at 4 °C until use.

### Preparation of Azido-Functionalized PEG-PDLLA Small Spherical and Tubular Polymersomes (N_3_-SmS and N_3_-SmT)

In order to down-size the assembled polymersomes, the preparation procedure was slightly adjusted. First, to increase membrane flexibility for extrusion (down-sizing process), polymersomes were assembled by adding 1 mL (33 vol %) of endotoxin-free water at a rate of 1 mL/h to the co-polymer solution in organic solvent (10 mg/mL co-polymer in 2 mL dioxane/THF 4:1 v/v). Prior to dialysis, the polymersome solution was extruded by passing the solution 11 times through an Avanti Mini-Extruder, which was assembled with a 100 nm polycarbonate membrane filter supported by two 10 mm filter supports. All materials of the extrusion set were extensively washed with endotoxin-free water prior to use.

### Analysis of Polymer Concentration

GPC was used to determine the polymer concentration in all polymersome samples. Based on the acquired data, samples were diluted so that an equal polymer concentration (of 2.3 mg/mL) across all different samples was obtained. A calibration curve was prepared by injection of 0, 1, 3, 5, 10, and 30 μL polymer stock solution (95 wt % PEG_24_-PDLLA_47_**3** with 5 wt % N_3_-PEG_24_-PDLLA_45_**5**, 2 mg/mL in THF, filtered through a 0.2 μm PTFE filter). Measurements were performed on lyophilized polymersome samples in THF (30 μL of 0.25 mg/mL co-polymer)—all samples were prepared in triplicate. Polymersome lyophilization was performed in a 1.5 mL Eppendorf tube. After lyophilization, 500 μL of THF was added and vortexed for 5 min to dissolve the polymer. Then, the mixture was centrifuged for 5 min at 13,000 × *g* to pellet the PBS salts. The supernatant was carefully removed with a 1 mL syringe capped with a needle and subsequently filtered through a 0.2 μm PFTE filter. Acquired data were analyzed using LC solutions software, by determining the area under the curve at an absorption wavelength of 218 nm for both standards and samples to plot a standard curve (Figure S3), allowing accurate determination of the polymer concentration.

### Dynamic Light Scattering (DLS) and Zeta (ζ) Potential Measurements

DLS and zeta potential measurements were performed using a Malvern instrument Zetasizer (model Nano ZSP). Zetasizer software was used to process and analyze the data. DLS measurements were conducted at 25 °C using a ZEN0040 type disposable cuvette cell (100 μL diluted sample volume). Azido-polymersomes (100 μL, 2.3 mg/mL polymer) were 10-fold diluted with Milli-Q water (900 μL). For DLS measurements of azido-polymersomes, an average of three measurements (10 scans per measurement at attenuator 6) was used to analyze the hydrodynamic diameter (*D*_h_ = *Z*-average diameter) and distribution (PDI). ζ Potential measurements of azido-polymersomes were conducted at 25 °C and 150 V using a DTS1070 folded capillary cell (900 μL diluted sample volume). An average of three measurements, with intervals of 2 min, was used to calculate the final ζ potential. Zeta deviation was reported as the standard deviation. For DLS measurements of aAPCs, samples were also diluted 10-fold, in PBS. Every aAPC topology was measured once (10 scans per measurement at attenuator 6), and the average of 15 topologies was used to analyze the mean size (*Z*-average diameter) and distribution (PDI) per morphology.

### Cryogenic Transmission Electron Microscopy (cryo-TEM)

Experiments were performed on the TU/e cryoTITAN (Thermo Fisher Scientific) operated at 300 kV equipped with a field emission gun. Grids with R 2/2 holey carbon film (Cu 200-mesh grids, Quantifoil Micro Tools GmbH, part of the SPT Life Sciences group) for cryo-TEM measurements were first plasma treated in a Cressington 208 carbon coater for 40 s before being used. Then, 3 μL of the polymersome solution was pipetted on the grid and blotted in a Vitrobot MARK IV (Thermo Fisher Scientific) at 100% humidity. The grid was blotted for 3.5 s (offset −3) and directly plunged and vitrified in liquid ethane. Processing of TEM images was performed with ImageJ, a program developed by NIH and available as public domain software at http://rsbweb.nih.gov/ij/. The nanotube aspect ratio was calculated by dividing the measured length by the width of each tube and calculating the mean value.

### Formation of aAPCs

In order to form polymersome-based aAPCs, DBCO-functionalized and labeled antibodies (αCD3-DBCO-ATTO488 and αCD28-DBCO-AF647, see the Supporting Information for the antibody functionalization method) were covalently conjugated to azido-polymersomes via a SPAAC reaction. To prepare monofunctional aAPCs, either αCD3 or αCD28 was added, whereas, for the preparation of bifunctional aAPCs, αCD3 and αCD28 were added in a 1:2 ratio. The initial concentration of reacting antibodies was varied so that aAPCs with various antibody densities could be prepared. Hereto, a series of decreasing concentrations of DBCO-labeled antibody solutions in PBS (12, 6, 3, 1.5, 0.75, and 0 μM) was added (100 μL for αCD3 and/or 200 μL for αCD28) to endotoxin-free PBS in Eppendorf LoBind microcentrifuge tubes. Next, 200 μL of the azido-polymersomes (polymer concentration = 2.3 mg/mL) was added to the antibodies; the N_3_-PEG-PDLLA concentration was calculated to be 8 μM on the polymersome surface. All Eppendorf tubes contained a final volume of 1200 μL to normalize the reaction volume and lower the antibody concentration to prevent cross-linking during the SPAAC click reaction. The concentration during the reaction was 1, 0.5, 0.25, 0.13, 0.063, and 0 μM for αCD3 and/or 2, 1, 0.5, 0.25, 0.13, and 0 μM for αCD28. For the bifunctional polymersome conjugates, αCD3 and αCD28 were added in a 1:2 ratio. For both monofunctional and bifunctional aAPCs, the polymer concentration was 0.4 mg/mL, with a N_3_-PEG-PDLLA concentration of 1.3 μM; the final ratio of αCD3/N_3_ was therefore 0.8, 0.4, 0.2, 0.1, and 0.05, and that of αCD28/N_3_ was 1.6, 0.8, 0.4, 0.2, and 0.1. The reaction mixture was incubated for 2 h at 30 °C on a thermoshaker at 300 rpm (ThermoMixer C with SmartBlock, 14-285-562PM), followed by overnight incubation on a tube rotator (Thermo Scientific Tube Revolver/Rotator #88881001) at a controlled temperature of 16 °C in a fridge. After conjugation, the polymersome conjugates were purified using centrifugation, as described below.

### General Procedure for aAPC Purification

After conjugation, N_3_-PEG_3_-NH_2_ (100 μL, 36 mM) was added to the polymersome/antibody reaction mixture to quench reactive DBCO groups on the unreacted (free) antibodies. The reaction mixture was incubated for 10 min on a tube rotator. Then, the reaction mixture was centrifuged (18,000 × *g*) for 30 min at 4 °C (Eppendorf Refrigerated 5424R Microcentrifuge, FA-45-24-11) to pellet the aAPCs. The supernatant—containing free antibodies—was removed, and the pellet was washed by resuspension in 1000 μL of 0.1% Tween-20 endotoxin-free PBS (0.1% EF-PBST), followed by vortexing for 10 s. Thereafter, both the centrifugation and washing steps were repeated, once with 0.1% endotoxin-free PBST and three times with endotoxin-free PBS. After the last round of centrifugation, 1000 μL of the supernatant was removed and purified aAPCs were resuspended in endotoxin-free PBS. Purified aAPCs of two individual experiments were combined and concentrated to a final volume of 500 μL (30 min, 18,000 × *g*, 4 °C). These aAPCs were used for final antibody quantification, characterization, and *in vitro* studies.

### Antibody Quantification

Concentrations of fluorophore-labeled antibodies on aAPCs were determined using a Tecan Spark 10 M fluorescence plate reader. For these measurements, all samples were loaded in a Thermo Fisher Scientific Nunclon 384 Flat Black plate. aAPCs were 10-fold diluted with endotoxin-free PBS buffer. To calculate the concentration of conjugated antibodies, a calibration curve of fluorescence intensity versus antibody concentration was used (Figure S9). All samples and standards were measured in triplicate, with a final volume of 50 μL per well. Plates were centrifuged (1000 × *g*, 1 min) prior to measurements to remove air bubbles and ensure optimal and similar positioning of all samples at the bottom of every well. ATTO488 and AF647 were excited at 485 and 635 nm, and emission was detected at 535 and 685 nm (bandwidth 20 nm), respectively.

### Stochastic Optical Reconstruction Microscopy (STORM)

To obtain high resolution STORM images, aAPCs were immobilized in a glass coverslip chamber, assembled using a glass coverslip (22 mm × 22 mm, thickness #1,5), and mounted on a glass microscopy slide separated by double-sided tape. To promote attachment of aAPCs to the coverslip surface, the chamber was incubated for 10 min with 0.1% poly-l-lysine and washed with PBS. aAPCs were incubated in the chamber overnight at 4 °C in a humidity chamber, and unbound structures were removed by washing the chamber with PBS. Before STORM imaging, PBS buffer was replaced with STORM buffer containing PBS, an oxygen-scavenging system (0.5 mg/mL glucose oxidase, 40 μg/mL catalase, and 5% w/v glucose), and 100 mM cysteamine. STORM images were acquired using a Nikon N-STORM system configured for total internal reflection fluorescence (TIRF) imaging. Two color STORM images were obtained by sequential imaging of dye-labeled antibodies on the aAPCs. AlexaFluor647-labeled antibodies were imaged first by illuminating the sample with the 647 nm laser line, followed by the imaging of ATTO488-labeled antibodies using the 488 nm laser line. Fluorescence was collected using a Nikon 100×, 1.4 NA oil immersion objective and passed through a quad-band-pass dichroic filter (97335 Nikon). Images were acquired by an EMCC camera (iXon3, Andor) at 16 ms integration time and a total of 15,000 frames per channel. STORM images were analyzed using the STORM module of the NIS elements software (Nikon) and ImageJ. STORM localizations were filtered to remove background by applying a density filter threshold of minimum 30 localizations in a 50 nm radius for spherical aAPCs and 30 localizations in a 200 nm radius for tubular aAPCs.

### Pan T Cell Isolation

Peripheral blood mononuclear cells (PBMCs) were isolated from healthy donor-derived buffy coats (Sanquin, The Netherlands) by density gradient centrifugation (Lymphoprep; STEMCELL, Canada). In advance, informed consent was obtained from every individual blood donor. T cells were isolated from PBMCs using the Pan T Cell Isolation Kit (Miltenyi Biotec, Germany) according to its protocol, which involved a negative selection of T cells by magnetic-activated cell sorting (MACS). Cells expressing CD14, CD15, CD16, CD19, CD34, CD36, CD56, CD123, and CD235a were magnetically labeled and subsequently depleted. Isolation efficiency and sample purity were assessed by sampling from cell populations before (PBMCs) and after (enriched and depleted cells) isolation for flow cytometric analysis of CD2 and CD3 co-expression as phenotypic markers for T cells.

### CellTrace Violet Staining

For cell proliferation studies, purified T cells were stained with CellTrace Violet (CTV; ThermoFisher) by incubating 1 × 10^6^ cells/mL (in PBS with 1% fetal bovine serum, FBS) with CTV (5 μM; in PBS) at equal volume (1:1 v/v) for 10 min at 37 °C/5% CO_2_ before an equal volume (1:1:1 v/v/v) of FBS was added. T cells were incubated for 30 min at 37 °C/5% CO_2_ and washed twice with HS-supplemented (2%; Sanquin) X-VIVO medium (Lonza Bioscience, Switzerland).

### T Cell Activation Assays

To examine the effects of aAPC signal density and size and shape, 5 × 10^4^ cells/well in a 96-well round-bottom plate were stimulated with aAPCs or soluble αCD3 and αCD28 in two independent experiments (*N* = 3 donors per exp.). Dynabeads (bead 1:1 cell; ThermoFisher) and empty polymersomes (at comparable particle concentrations) were included as positive and negative controls, respectively. T cells were cultured in X-VIVO/2% HS at 37 °C/5% CO_2_ for either 6 or 24 h at 1–500 ng/mL (exp. 1) or 1 or 3 days at 25–125 ng/mL (exp. 2) total αCD3. A concentration of 1–500 ng/mL contained approximately (6–10) × 10^3^ large aAPCs or (24–40) × 10^3^ small aAPCs per T cell. First (exp. 1), the expression of CD69 and CD25 was determined with flow cytometry and the production of IL-2 and IFNγ was measured with ELISA (*n* = 2 repl.). Second (exp. 2), the expression of CD25 and PD-1 was determined with flow cytometry and the production of IL-2 and IFNγ was measured with ELISA.

### ELISA Procedures

Cytokine concentrations in supernatants obtained from T cell cultures were measured with commercially available kits (IFN gamma Human Uncoated ELISA Kit and IL-2 Human Uncoated ELISA Kit, both ThermoFisher). Standards and samples were measured in duplicate, and samples were appropriately diluted to fall within the calibration curve range. Values of IL-2 and IFNγ were normalized to response after stimulation with 125 ng/mL soluble αCD3 and αCD28.

### Flow Cytometry

For flow cytometric analysis, the following antibodies were purchased from BioLegend: CD2-BV510 (clone RPA-2.10), CD3-FITC (clone HIT3a), CD4-APC/Cy7 (clone RPA-T4), CD8-PE/Cy7 (clone SK1), CD25-PE (clone M-A251), CD69-PE/Cy5 (clone FN50), PD-1-PerCP/Cy5.5 (clone EH12.2H7). Dead cells were stained using the Zombie Violet Fixable Viability Kit (BioLegend). Staining was performed in PBS or PBS supplemented with 1% BSA and T cells were fixed in FluoroFix Buffer (BioLegend) before flow cytometric acquisition. Fluorescence was measured using a CyAn ADP Analyzer instrument (Beckman Coulter, The Netherlands) and data was analyzed using FlowJo (version 10.7.1, Tree Star, Inc., USA) to obtain mean fluorescence intensity (MFI; geometric mean), population frequencies, and proliferation data (division index, average number of cell divisions). Normalized MFI (NMFI) was calculated by multiplying the frequency of positive cells by the MFI within the positive population.

### Statistical Analysis

Data are presented as the mean ± standard error (SE) or ± standard deviation (SD). All *p*-values were two-tailed, and *p* < 0.05 was considered significant. The family-wise error rate (FWER) was protected by using an extra sum-of-squares F test or a one-way or two-way analysis of variance (ANOVA). In the case of rejection of the overall test, conditions were compared pairwise to investigate where the difference originated from. Multiplicity adjusted *p*-values were calculated using the Bonferroni or Tukey correction for multiple comparison. Sigmoidal dose–response curves were fitted through nonlinear regression using a variable slope model (four-parameter logistic curve). Statistical tests were performed by using Prism 9.0.1 software (GraphPad Software, Inc., USA).

## Supporting Information Available

Polymer synthesis and characterization, antibody functionalization and analysis, polymersome characterization, conjugation methodology and aAPC library analysis, flow cytometry gating strategies, and T cell activation data (PDF)

## Abbreviations

aAPC: artificial antigen-presenting cell
ACT: adoptive cell transfer
ANOVA: analysis of variance
CD: cluster of differentiation
cryo-TEM: cryogenic transmission electron microscopy
cSMAC: central supramolecular activation cluster
CTV: CellTrace Violet
DC: dendritic cell
DLS: dynamic light scattering
ELISA: enzyme-linked immunosorbent assay
HD: high density
ID: intermediate density
IFN: interferon
IL: interleukin
IS: immunological synapse
LgS: large spheres
LgT: large tubes
LD: low density
NMFI: normalized mean fluorescence intensity
NTA: nanoparticle tracking analysis
PD-1: programmed cell death protein 1
PEG-PDLLA: poly(ethylene glycol)-*block*-poly(d,l-lactide)
PIC: polyisocyanopeptide
pSMAC: peripheral supramolecular activation cluster
SD: standard deviation
SE: standard error
SmS: small spheres
SmT: small tubes
STORM: **s**tochastic optical reconstruction microscopy
TCR: T cell receptor
