# Supplementary material for: Artificial Antigen-Presenting Cell Topology Dictates T Cell Activation
Source: ACS Nano. 2022 Aug 15;16(9):15072–85. doi: 10.1021/acsnano.2c06211 (PMC9527792; doi:10.1021/acsnano.2c06211)
Supplement: Supplementary file 1 — nn2c06211_si_001.pdf [file nn2c06211_si_001.pdf]

Supporting Information

Artificial Antigen-Presenting Cell Topology Dictates T cell Activation

Annelies C. Wauters[1,2,3], Jari F. Scheerstra[1], Irma G. Vermeijlen[1], Roel Hammink[2,3], Marjolein Schluck[2,3], Laura Woythe[4], Hanglong Wu[1], Lorenzo Albertazzi[4,5], Carl G. Figdor[2,3,6], Jurjen Tel[4,7], Loai K. E. A. Abdelmohsen[1], Jan C. M. van Hest[1*].*

[1] Bio-Organic Chemistry, Institute for Complex Molecular Systems (ICMS), Eindhoven University of Technology, 5600 MB Eindhoven, The Netherlands.

[2] Department of Tumor Immunology, Radboud Institute for Molecular Life Sciences, Radboud University Medical Center, 6525 GA Nijmegen, The Netherlands.

[3] Division of Immunotherapy, Oncode Institute, Radboud University Medical Center, 6525 GA Nijmegen, The Netherlands.

[4] Department of Biomedical Engineering, Institute of Complex Molecular Systems (ICMS), Eindhoven University of Technology, 5600 MB Eindhoven, The Netherlands.

[5] Institute for Bioengineering of Catalonia (IBEC), The Barcelona Institute of Science and Technology (BIST), Barcelona 08036, Spain.

[6] Institute for Chemical Immunology, 6525 GA Nijmegen, The Netherlands.

[7] Laboratory of Immunoengineering, Department of Biomedical Engineering, Eindhoven University of Technology, 5600 MB Eindhoven, The Netherlands.

CONTENTS

SUPPLEMENTARY INSTRUMENTS

SUPPLEMENTARY METHODS

SUPPLEMENTARY TABLE 1

SUPPLEMENTARY FIGURES 1-20

REFERENCES

SUPPLEMENTARY INSTRUMENTS

Nuclear Magnetic Resonance (NMR). Proton nuclear magnetic resonance (^{1}H NMR) measurements were performed on a Bruker 400 Ultrashield™ spectrometer equipped with a Bruker SampleCase autosampler, using $CDCl_3$ as a solvent and TMS as an internal standard. For these measurements, block copolymers were dissolved at a concentration of 10 mg/mL. The obtained spectra were analyzed using MestReNova NMR analysis software.

Gel permeation chromatography (GPC). GPC was conducted using a Shimadzu Prominence-i GPC system with a PL gel 5 µm mixed D and mixed C column (Polymer Laboratories) and a Shimadzu RID-20A differential refractive index detector. THF was used as an eluent with a flow rate of 1 mL/min. Block copolymers were dissolved in THF at a concentration of 1 mg/mL before conducting the measurements.

Differential scanning calorimetry (DSC). To determine the glass transition temperature (T_g) of the block copolymers, DSC data were collected on a DSC Q2000 from TA instruments, calibrated with an indium standard. The lyophilized copolymers (4–8 mg) were weighed directly into aluminum pans and hermetically sealed. The samples were initially heated to 20°C and then subjected to a heating and cooling cycle to 80°C and -20°C, respectively, with a scanning rate of 5°C/min. This was followed by three cooling/heating cycles from -20°C to 80°C with a scanning rate of 5, 10 and 40°C/min, respectively. The T_g was determined at the inflection point from the last heating/cooling cycle.

Infrared spectroscopy (IR). IR spectra of lyophilized copolymers were recorded on a PerkinElmer Spectrum One spectrometer.

UV/Vis spectroscopy. The UV/Vis spectrophotometer measurements were performed on a NanoDrop® ND-1000 UV-Vis Spectrophotometer (Thermo Fisher Scientific).

Fluorescence spectrometry. Fluorescence intensity measurements were performed on a Tecan Spark 10M multimode plate reader.

Nanoparticle tracking analysis (NTA). To track the number of particles and measure their hydrodynamic size distributions, a Nanosight NS300 instrument (Malvern Panalytical) equipped with a scientific complementary metal–oxide–semiconductor (sCMOS) camera was used. The camera was mounted on an optical microscope, allowing visualization of the light scattered by the injected particles that were present in the focus of an 80 µm beam generated by a single mode laser diode with a blue laser (488 nm). Large and small aAPCs were diluted approximately 200 and 700-fold in PBS, respectively, so that the number of particles in the field of view was in the recommended range of 20-100, and a volume of 1 mL was injected into the Nanosight chamber. For all measurements 3 captures of 30 sec were recorded, with a screen gain of 9 and a camera level of 11; during the data analysis the screen gain was set to 9 with a detection threshold of 5. Particle number concentrations and surface area concentrations were determined from the area under the curve of the size distribution with number weighting or surface area weighting, respectively.

SUPPLEMENTARY METHODS

Synthesis and characterization of block copolymer poly(ethylene glycol) – poly(D,L-lactide) (PEG_{22}-$PDLLA_{47}$ 3). The synthesis of PEG_{22}-$PDLLA_{47}$ **3** was performed according to our previously reported procedure[1–3] (synthesis scheme is outlined in **Figure S2a**). A round bottom flask (50 mL) equipped with a stirring bar was dried using a heat gun under vacuum, followed by three cycles of flushing with argon and evacuation. Thereafter, the flask was subjected to a constant flow of argon. Monomethoxy-PEG-OH **1** (mPEG, 0.194 g, 0.2 mmol, poly(ethylene glycol) 1K, JenKem technology, lyophilized) and D,L-Lactide **2** (DLL, 1.3 g, 9 mmol, Acros) were added to the dried flask. Subsequently, dry toluene (ca. 50 mL) was added using an argon-flushed syringe and the solvent was evaporated to dry the reagents. The rotary evaporator was flushed with an argon balloon after solvent evaporation in order to maintain an inert atmosphere after drying, and this toluene evaporation cycle was repeated once more. The dried reagents were re-dissolved in dry DCM (13 mL; [DLL monomer] = 0.7M) using an argon-flushed syringe before DBU (15 µL, 0.5 equivalents relative to the amount of mPEG macroinitiator) was added using an argon-flushed organic solvent pipette. The reaction was stirred at 25°C for 2 hours until ^{1}H NMR analysis showed full conversion of the D,L-lactide monomer. Subsequently, the reaction mixture was diluted with DCM (25 mL) and washed twice with 1M $KHSO_4$, once with Milli-Q water, and once with brine (100 mL each). The organic solution was dried using Na_2SO_4. After evaporating most of the solvent, the concentrated copolymer solution was precipitated into ice-cold diethyl ether (200 mL). The resulting waxy solid was partially dried under argon, dissolved in dioxane, and lyophilized to yield a white powder (yield = 1.2 gram, ~80% recovered polymer). The synthesized copolymer was characterized using ^{1}H NMR, GPC and DSC to determine copolymer composition, polydispersity and glass transition temperature (T_g), respectively (**Figure S2c,e,f**). ^{1}H NMR ($CDCl_3$, 400 MHz, δ in ppm): 5.18 (dq, 95H, lactide CH, *a*), 3.64 (s, 88H, PEG backbone, *c*), 3.38 (s, 3H, Me, *d*), 1.57 (m, 283H, lactide CH_3, *b*) (**Figure S2c**). GPC: Đ = 1.1 (**Figure S2e**). DSC: T_g = 25°C (**Figure S2f**).

Synthesis and characterization of block copolymer N_3-PEG_{24}-$PDLLA_{45}$ 5. Using a procedure similar to the ring-opening polymerization described for PEG_{22}-$PDLLA_{45}$ **3**, azido-dPEG^{TM}(24)-OH **4** (220 mg, 0.2 mmol, Iris Biotech GmbH) and D,L-lactide **2** (1300 mg, 9 mmol) were used to synthesize azido-functionalized block copolymer N_3-PEG_{24}-$PDLLA_{45}$ **5** (synthesis scheme is outlined in **Figure S2b**), yielding a white powder (yield = 1.0 g, ~70% recovered polymer). ^{1}H-NMR ($CDCl_3$, 400 MHz, δ in ppm): 5.18 (m, 90H, lactide CH, *a*), 3.72 – 3.57 (m, 94H, PEG backbone, *c*), 3.39 (t, 2H, CH_2-N_3, *r*), 1.61 – 1.49 (m, 270H, m, lactide CH_3, *b*), see **Figure S2d** for spectrum. GPC analysis revealed a Đ of 1.3 (**Figure S2e**). DSC analysis revealed a T_g of 18°C (**Figure S2f**). IR analysis showed absorption bands at characteristic wavenumbers for functional groups including hydroxyl, alkanes and esters. Of particular interest is the absorption band that was observed at wavenumber 2107 cm^{-1}, which is characteristic for the azido-functionality (**Figure S2g**).

Antibody functionalization and analysis. For functionalization, about 2-5 mg of αCD3 (OKT3 clone, bioXcell) or αCD28 (9.3 clone, bioXcell) were buffer exchanged to borate buffer (pH8.4, 50 mM) to final concentrations of about 2-3 mg/ml. Next, DBCO-PEG4-NHS (4 eq.) and ATTO488-NHS (2 eq., αCD3) or AlexaFluor647-NHS (2 eq, αCD28) were added and the reaction was incubated for 2-4 hours at 4°C. After incubation, we purified the antibodies using Zeba spin columns (Thermofisher, 5 ml, 40 kDa MWCO) using the manufacturer's protocol, with the exception that 5 washes instead of 3 were used.

UV-Vis spectrophotometer measurements were performed to determine the degree of DBCO and fluorophore labeling on the functionalized antibodies, as well as the antibody concentration. The system was equilibrated using 2 µL of Milli-Q water, followed by 2 µL of PBS buffer as a background blank. Then, 2 µL of sample was pipetted into the spectrophotometer and the absorbance spectrum was measured. To calculate the concentrations of antibodies, DBCO, ATTO488 and AF647, the absorbance was measured at 280, 309, 504, and 651 nm, respectively. All extinction coefficients and used formulas to correct for the spectral overlap of the individual molecules and calculate the respective concentrations are described in the equations and the table below.

Abbreviation	Explanation	Value
A_{c280}, A_{c309}	Corrected absorbance at 280, 309 nm	-
$A_{280}, A_{309}, A_{504}, A_{651}$	Absorbance signal measured at 280, 309, 504, 651 nm	-
$M_{\alpha CD3}, M_{\alpha CD28}, M_{DBCO}, M_{ATTO488}, M_{AF647}$	Molar concentration in mol L^{-1} for the individual molecules	-
$C_{\alpha CD3}, C_{\alpha CD28}$	Antibody concentration in mg/mL	-
c_1	Correction factor A309 (DBCO) on A280 (protein), determined as A280/A309 of DBCO-PEG-NHS	0.880
c_2	Correction factor A280 (protein) on A309 (DBCO), determined as A309/A280 of non-functionalized antibody	0.028
c_3	Correction factor ATTO488 on A309 (DBCO), determined as A309/A504 of ATTO488-NHS	0.162
c_4	Correction factor ATTO488 on A280 (protein), determined as A280/A504 of ATTO488-NHS	0.050
c_5	Correction factor AF647 on A309 (DBCO), determined as A309/A651 of AF647-NHS	0.033
c_6	Correction factor AF647 on A280 (protein), determined as A280/A651 of AF647-NHS	0.030
$MW_{\alpha CD3}, MW_{\alpha CD28}$	Molecular weight antibody in kDa (kg/mol)	150
ε_{DBCO}	Extinction coefficient DBCO in $M^{-1}cm^{-1}$	12,000
$\varepsilon_{\alpha CD3}, \varepsilon_{\alpha CD28}$	Extinction coefficient antibodies in $M^{-1}cm^{-1}$	210,000
$\varepsilon_{ATTO488}$	Extinction coefficient ATTO488 in $M^{-1}cm^{-1}$	90,000
ε_{AF647}	Extinction coefficient AF647 in $M^{-1}cm^{-1}$	270,000
l	Optical path length in cm	0.1
$DOL_{DBCO}, DOL_{ATTO488}, DOL_{AF647}$	Degree of labeling of DBCO, ATTO488, AF647 on antibody	-

αCD3-ATTO488-DBCO:

$$A_{c280} = (A_{280} - A_{309} \times c_1 + A_{504} \times c_1 \times c_3 - A_{504} \times c_4)/(1 - c_1 \times c_2)$$
$$M_{\alpha CD3} = \frac{A_{c280}}{(\varepsilon_{\alpha CD3} \times l)}$$
$$C_{\alpha CD3} = M_{\alpha CD3} \times MW_{\alpha CD3}$$

$$A_{c309} = A_{309} - (A_{c280} \times c_2) - (A_{504} \times c_3)$$
$$M_{DBCO} = \frac{A_{c309}}{(\varepsilon_{DBCO} \times l)}$$
$$DOL_{DBCO} = M_{DBCO} / M_{\alpha CD3}$$

$$A_{c504} = A_{504}$$
$$M_{ATTO488} = \frac{A_{c504}}{(\varepsilon_{ATTO488} \times l)}$$
$$DOL_{ATTO488} = M_{504} / M_{280}$$

αCD28-AF647-DBCO:

$$A_{c280} = (A_{280} - A_{309} \times c_1 + A_{651} \times c_1 \times c_5 - A_{651} \times c_6)/(1 - c_1 \times c_2)$$
$$M_{\alpha CD28} = \frac{A_{c280}}{(\varepsilon_{\alpha CD28} \times l)}$$
$$C_{\alpha CD28} = M_{280} \times MW_{\alpha CD28}$$

$$A_{c309} = A_{309} - (A_{c280} \times c_2) - (A_{651} \times c_5)$$
$$M_{DBCO} = \frac{A_{c309}}{(\varepsilon_{DBCO} \times l)}$$
$$DOL_{DBCO} = M_{DBCO} / M_{\alpha CD28}$$

$$A_{c651} = A_{651}$$
$$M_{AF647} = \frac{A_{c651}}{(\varepsilon_{AF647} \times l)}$$
$$DOL_{AF647} = M_{AF647} / M_{\alpha CD28}$$

Theoretical estimations for antibody conjugation. Molar ratios between the antibodies and available reactive moieties on the polymersome surface were calculated. The maximum number of antibodies that could be retained on one particle (Ab_{max}) was theoretically estimated at 900 Abs/small polymersome and 3600 Abs/large polymersome, according to the following equation:

$Ab_{max} = area_{particle(sphere)}/area_{antibody(circle)} = (\pi \cdot d^2)/(\frac{1}{4} \cdot \pi \cdot s^2)$

with the minimal antibody spacing *s* estimated at 10 nm at complete coverage of the surface area of small and large polymersomes with diameters *d* of 150 and 300 nm, respectively.

The number of N_3-polymers (N_3) per small or large polymersome surface was estimated at approximately 3,000 and 30,000, respectively. This value was based on the following equation:

$N_3 = \frac{1}{2}$ (only outside of bilayer)$\cdot$0.05 (wt.% polymer)$\cdot(w_{polymersome}/mw_{N3\text{-}polymer})\cdot N_a$,

with $w_{polymersome}$ the weight per polymersome, determined by polymer concentration (2.3 mg/mL) / polymersome concentration ($1.5 \cdot 10^{12}$ or $1.5 \cdot 10^{11}$ particles/mL),

$mw_{N3\text{-}polymer}$ the molecular weight of the N_3-polymer (7.5 kg/mol),

N_a Avogadro's number ($6 \cdot 10^{23}$ · molecules/mol).

Due to the relatively low number of DBCO on the antibodies (DOL≈2, to prevent crosslinking), as well as their large size (resulting in steric hindrance) a moderate to low conjugation efficiency was expected. Taking these boundaries into account, an excess initial feed of antibodies to available N_3 moieties was used to reach Ab_{max}.

Calculations of antibody density, number and conjugation efficiency. The antibody spacing (average distance between two antibodies), the antibody number per particle, and the conjugation efficiency were calculated according to Equations 1-3.

(1) $$Antibody\ spacing\ (nm) = \sqrt{\frac{\left(\frac{Mean\ surface\ area\ concentration}{Antibody\ conjugated \times N_a}\right)}{\pi}} \times 2$$

(2) $$Antibody\ number/particle = \frac{Antibody\ conjugated \times N_a}{Mean\ particle\ concentration}$$

(3) $$Conjugation\ efficiency\ c.e.(\%) = \frac{Antibody\ conjugated \times V_{final}}{Initial\ antibody\ concentration \times V_{initial}} \times 100\%$$

Parameter	Explanation	Value/Unit
$Mean\ surface\ area\ concentration$	Determined by NTA	nm^2/mL
$Mean\ particle\ concentration$	Determined by NTA	Particles/mL
$Antibody\ conjugated$	Concentration of αCD3 or αCD28 on the aAPCs	mol/L
N_a	Avogadro's number	$6 \cdot 10^{23}$ 1/mol
$Initial\ antibody\ concentration$	Initial αCD3 or αCD28 concentration	mol/L
V_{final}	Final aAPC volume after purification	µL
$V_{initial}$	Initial aAPC sample volume in conjugation reaction	µL

Stability of aAPCs. X-VIVO™ medium (Lonza Bioscience, Switzerland) supplemented with human serum (HS; 2%; Sanquin) was added to each different aAPC morphology in a 1:1 (v/v) ratio so that the polymer concentration was approximately 1 mg/mL with 1% HS. The mixture was incubated at 37°C and 300 rpm (ThermoMixer C with SmartBlock, 14-285-562PM). Hydrodynamic size and PDI were monitored with DLS after 0, 1, 4, 8, 24, 48, and 72 hours of incubation. The samples were diluted (5x) with PBS prior to DLS measurement to minimize the effect of serum proteins, and measurements were performed in triplicate.

SUPPLEMENTARY TABLE

Supplementary Table 1. Characterization of antibody concentration and degrees of labeling (DOL).

**Functionalized antibody**	**Antibody concentration (mg/mL)**	**DOL fluorescent dye**	**DOL DBCO**
αCD3-ATTO488-DBCO	2.78	1.4	2.2
αCD28-AF647-DBCO	2.05	1.0	1.8

SUPPLEMENTARY FIGURES

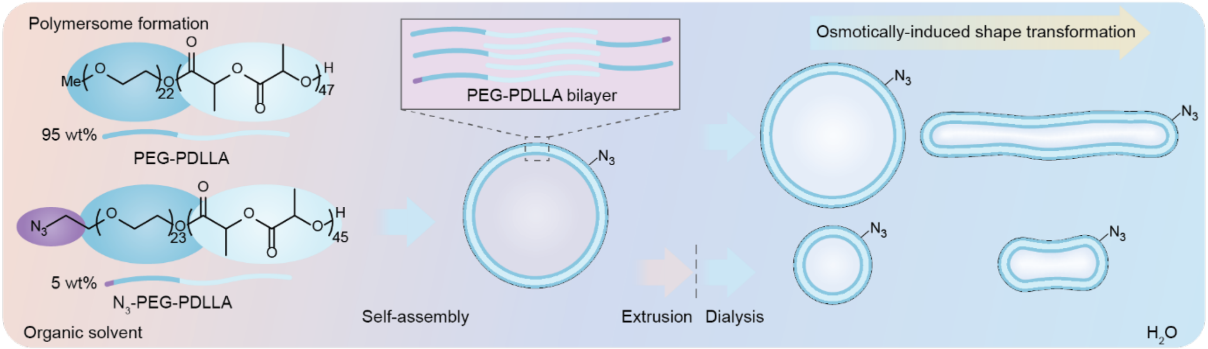

Figure S1. Formation of azido-polymersomes with controlled morphologies. To a mixture of PEG-PDLLA (95 wt.%) and N_3-PEG-PDLLA (5 wt.%) block copolymers in organic solvent, H_2O (50% or 33%) is added to induce self-assembly of N_3-polymersomes. To create large polymersomes, the polymersome solution (50% H_2O) is dialyzed to H_2O to form large spheres (N_3-LgS), or to 50 mM NaCl in H_2O to obtain large tubes (N_3-LgT) through osmotically-induced shape transformation. To create small polymersomes, the polymersome solution (33% H_2O) is extruded through a 100 nm membrane, prior to dialysis to H_2O or 50 mM NaCl, to form small spheres (N_3-SmS) or small tubes (N_3-SmT), respectively. Upon removal of the organic solvent plasticizer during dialysis, the polymersomes are trapped in their morphological structure.

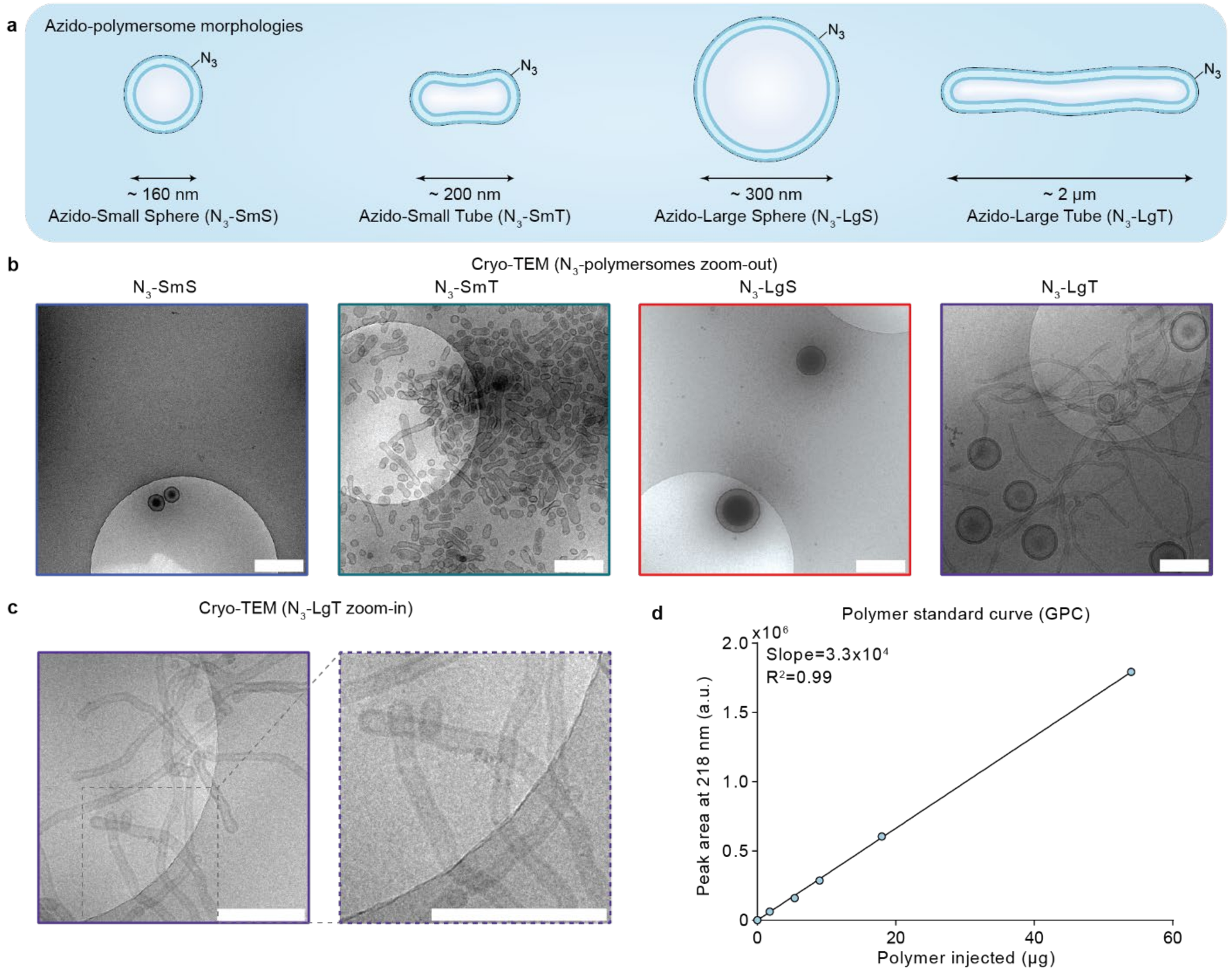

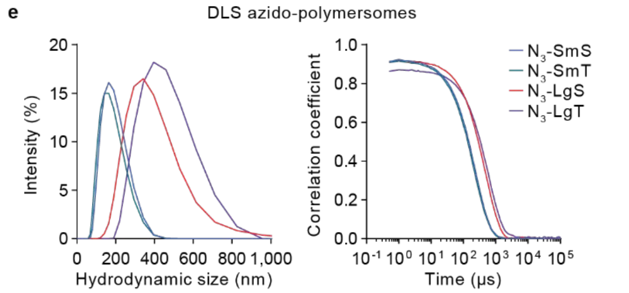

f Polymersome dimensions

Name	D_h (nm)	PDI	ζ potential (mV)	Length (nm)	Width (nm)	Aspect Ratio
**N_3-SmS**	161	0.09	-17 ±6	-	-	-
**N_3-SmT**	149	0.12	-17 ±6	229 ±12	67 ±14	3.7 ±2.2
**N_3-LgS**	322	0.09	-20 ±5	-	-	-
**N_3-LgT**	404	0.12	-21 ±5	1,862 ±860	46 ±15	41 ±18

Figure S2. Characterization of azido-polymersomes. Schematic illustration of the four azido-polymersome morphologies. **b**, Zoomed-out cryo-TEM images show the spherical or tubular morphology of the polymersomes. **c**, Zoomed-in cryo-TEM images of N_3-LgT show their tubular morphology. **d**, Polymer standard curve to determine polymer concentration. The area under the curve at 218 nm absorbance linearly correlates with different amounts of polymer injected, as measured with GPC. Cryo-TEM analysis was performed on spherical polymersomes in Milli-Q water or tubes in 50 mM NaCl. Scale bar = 500 nm. **e**, Dynamic light scattering (DLS) intensity profiles and correlation functions. **f**, Overview table of polymersome hydrodynamic diameters (D_h), zeta (ζ) potentials and dimensions based on DLS and cryo-TEM image analysis. Samples for DLS and ζ potential measurements were prepared in PBS, diluted ten-fold with Milli-Q water. Cryo-TEM measurements were performed on spheres in Milli-Q water and tubes in 50 mM NaCl. SmT: 89% tubes, n=40 tubes analyzed, LgT: 76% tubes (other particles are nested vesicles), n=20 tubes analyzed. Data represents mean ± SD.

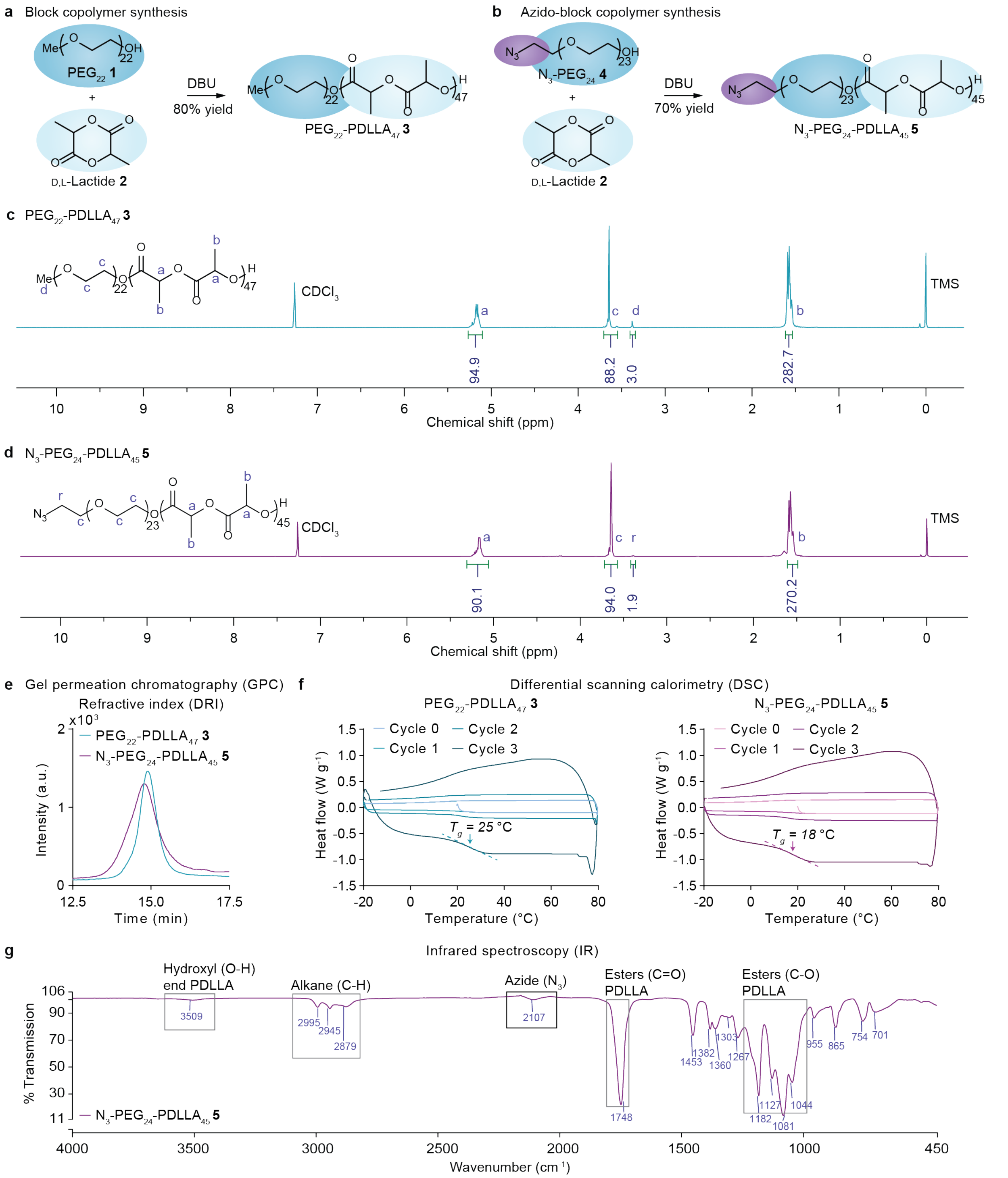

Figure S3. Block copolymer synthesis and characterization. **a**, DBU, DCM, 25°C, argon, 2 hours, 80% yield. **b**, DBU, DCM, 25°C, argon, 2 hours, 70% yield. **c**, ^{1}H NMR spectra ($CDCl_3$ with v/v 0.05% TMS, 400 MHz) confirming degree of polymerization of **3** and **5**. **d**, N_3-PEG_{24}-$PDLLA_{45}$ **5**. **e**, Gel permeation chromatography (GPC) refractive index (DRI) signals of polymers **3** and **5** indicating polydispersity ((Đ=1.1 and Đ=1.3, respectively). **f**, Differential scanning calorimetry (DSC) results of **3** and **5**, showing glass transition temperatures (T_gs) of 25°C and 18°C, respectively. **g**, Infrared spectroscopy (IR) spectra of **5** indicating the characteristic absorption band of the azido-moiety at 2107 cm^{-1}.

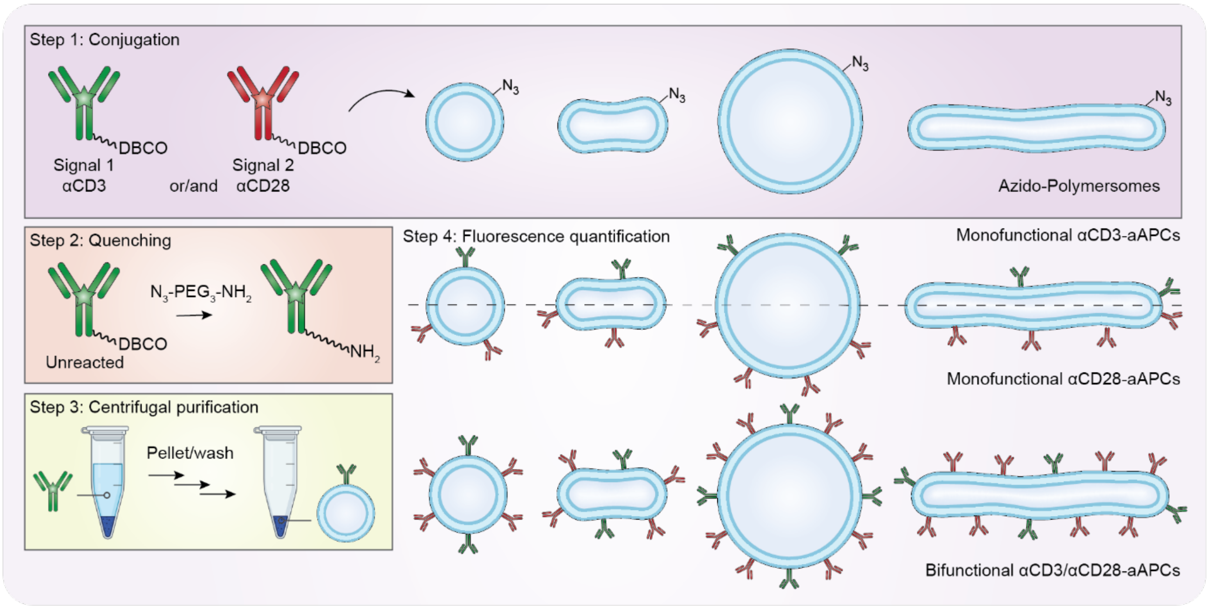

Figure S4. Engineering method for the development of an aAPC library with control over morphology and antibody density. A library with over 60 different aAPC topologies was developed in four steps, to control morphology (SmS, SmT, LgS, LgT), functionality (monofunctional αCD3 or αCD28, or bifunctional αCD3/αCD28) and density (five ligand densities). Step 1: Conjugation of cyclooctyne- and fluorescently labeled αCD3- or/and αCD28 antibodies to azido-polymersomes with various morphologies. Antibody:N_3-polymer ratios were varied to control ligand density. Step 2: Quenching of unreacted antibodies. Step 3: Effective purification of aAPCs through centrifugation. Step 4: Quantification of the conjugated αCD3 or/and αCD28 antibodies using a fluorescence spectroscopy microplate assay.

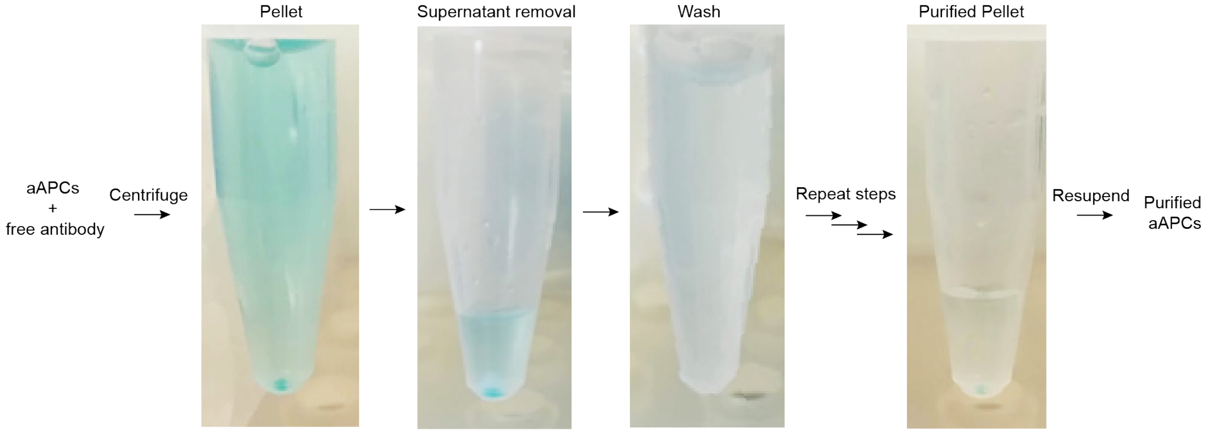

Figure S5. Centrifugal purification of aAPCs. Non-purified aAPCs with free antibodies are centrifuged to form a pellet of the aAPCs. The supernatant containing free antibodies is removed, and the pellet is subsequently washed through resuspension in fresh buffer. These steps are subsequently repeated until the purified aAPCs are obtained.

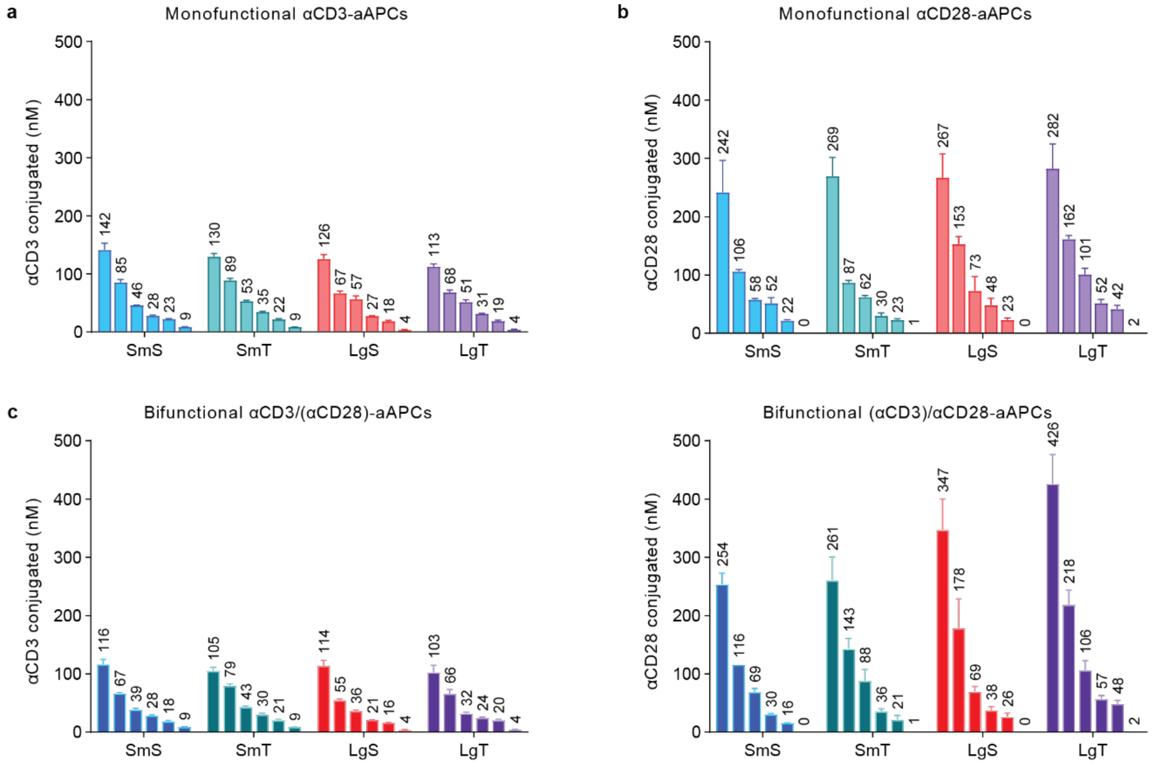

Figure S6. Quantification of antibody concentrations on aAPCs shows consistency for various topologies. The six bars per morphology indicate conjugate concentrations at different initial feed ratio of antibodies (αCD3:N_3 was 0.8, 0.4, 0.2, 0.1, 0.05, and 0; αCD28:N_3 was 1.6, 0.8, 0.4, 0.2, 0.1, and 0). Quantification of the concentration of conjugated (**a**) αCD3 for monofunctional αCD3-aAPCs, (**b**) αCD28 for monofunctional αCD28-aAPCs and (**c**) αCD3 (left) and αCD28 (right) for bifunctional αCD3/αCD28-aAPCs. Increased αCD3:αCD28 ratios for LgS and LgT might be attributed to variations in antibody stock solutions and pipetting. Data represents mean ± SD.

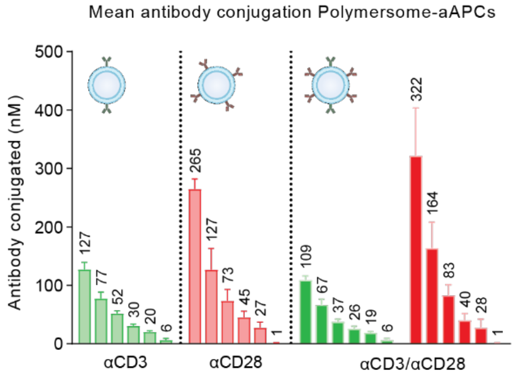

Figure S7. Mean antibody concentrations for monofunctional and bifunctional aAPCs. Average concentrations were calculated for four polymersome morphologies. The six bars indicate conjugate concentrations at different initial feed ratio of antibodies (αCD3:N_3 was 0.8, 0.4, 0.2, 0.1, 0.05 and 0; αCD28:N_3 was 1.6, 0.8, 0.4, 0.2, 0.1 and 0). Data represents mean ± SD (n=3, N=4).

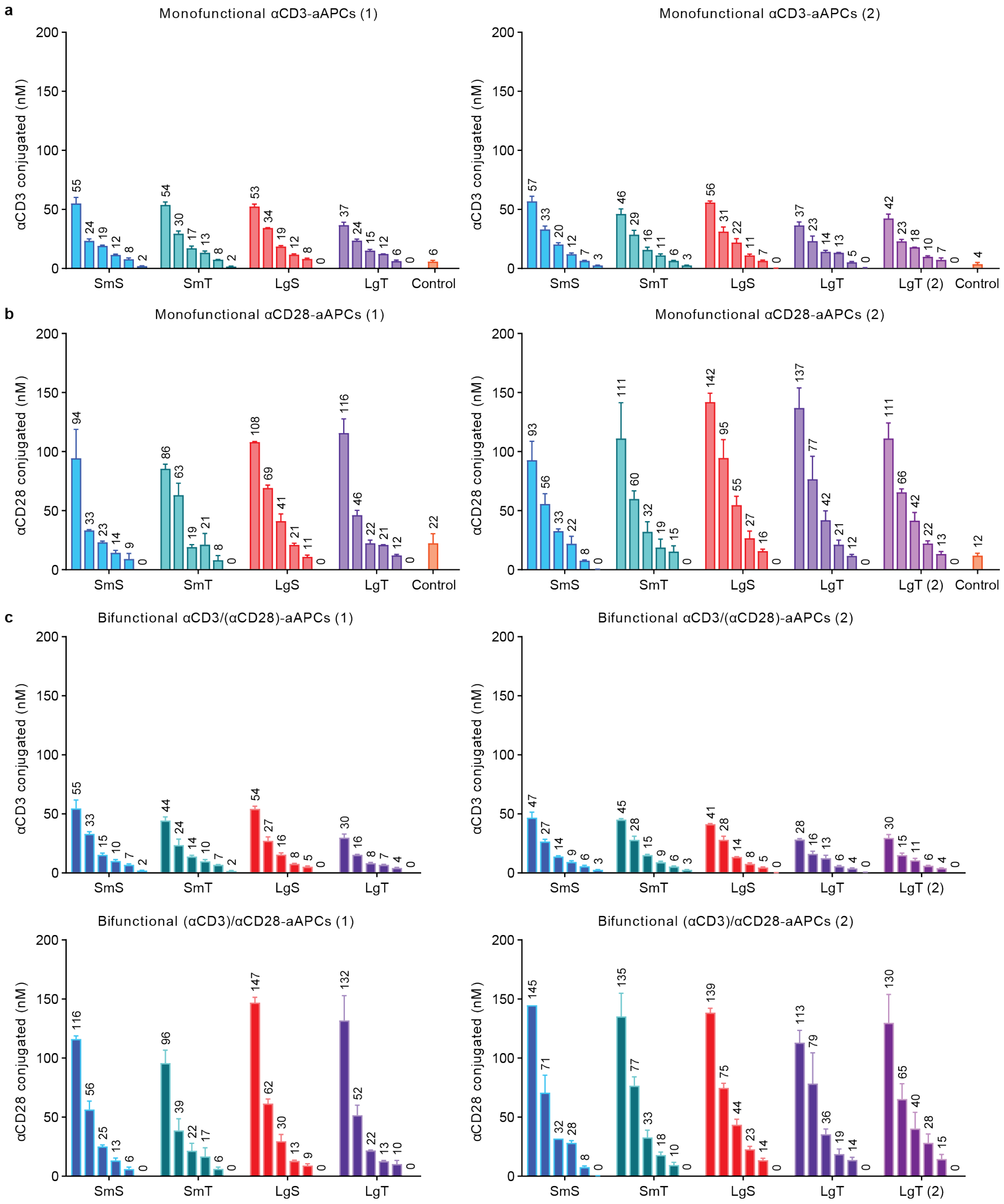

Figure S8. Antibody quantification for two independent experiments shows high reproducibility. Initial conjugation experiments, were performed in duplicate (1 and 2), which were later combined. The six bars per morphology indicate conjugate concentrations at different initial feed ratio of antibodies (αCD3:N_3 was 0.8, 0.4, 0.2, 0.1, 0.05, and 0; αCD28:N_3 was 1.6, 0.8, 0.4, 0.2, 0.1, and 0). Quantification of the concentration conjugated (**a**) αCD3 for monofunctional αCD3-aAPCs, (**b**) αCD28 for monofunctional αCD28-aAPCs and (**c**) αCD3 and αCD28 for bifunctional αCD3/αCD28-aAPCs of experiments (1) and (2). Controls (LgS without N_3-moieties incubated with the highest antibody concentration), indicate a relative nonspecific bound antibody concentration of approximately 10%, compared to those bound to N_3-polymersome-based aAPCs, indicating that 90% of unconjugated antibodies were removed. Data represents mean ± SD (n=3).

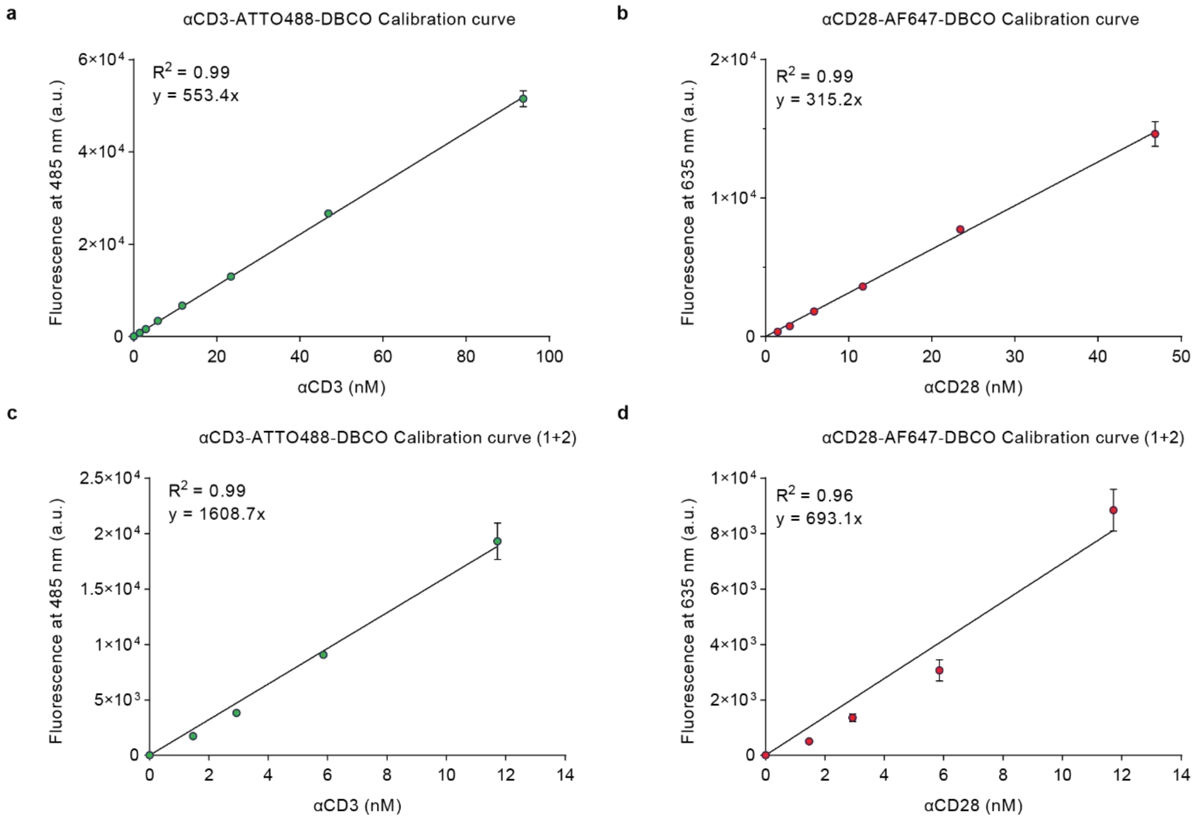

Figure S9. Antibody calibration curves used for antibody quantification using fluorescence spectroscopy. **a**, αCD3 calibration curve used for final quantification. **b**, αCD28 calibration curve used for final quantification. **c**, αCD3 calibration curve used for two individual experiments (1) and (2). **d**, αCD28 calibration curve used for two individual experiments (1) and (2). Data represents mean ± SD (n=3).

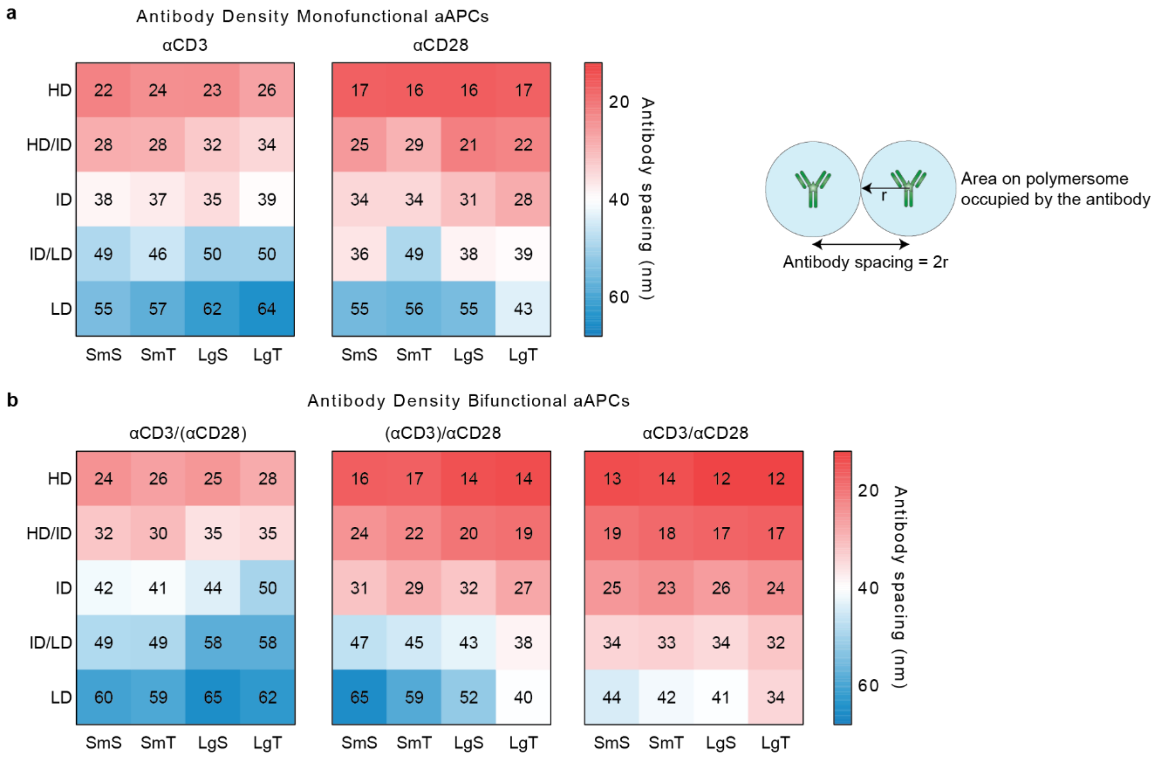

Figure S10. Quantification of antibody densities for different aAPC topologies. Heat maps indicating the antibody spacing (in nm) are organized by density (HD, ID, LD) and morphology (SmS, SmT, LgS, LgT). **a**, αCD3 and αCD28 spacings for monofunctional αCD3- and αCD28-aAPCs, respectively. **b**, αCD3, αCD28 and the total antibody spacings for bifunctional αCD3/αCD28-aAPCs. HD, ID, LD = high, intermediate, low density.

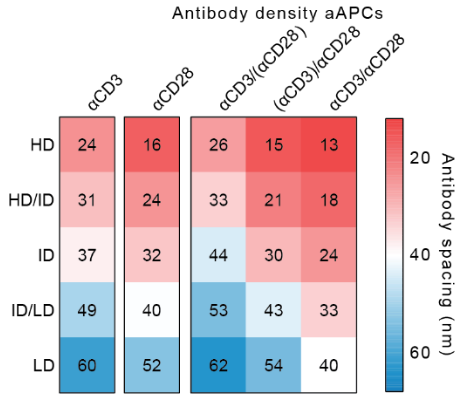

Figure S11. Mean antibody spacings for monofunctional and bifunctional aAPCs with different densities. Mean spacings were calculated for four polymersome morphologies.

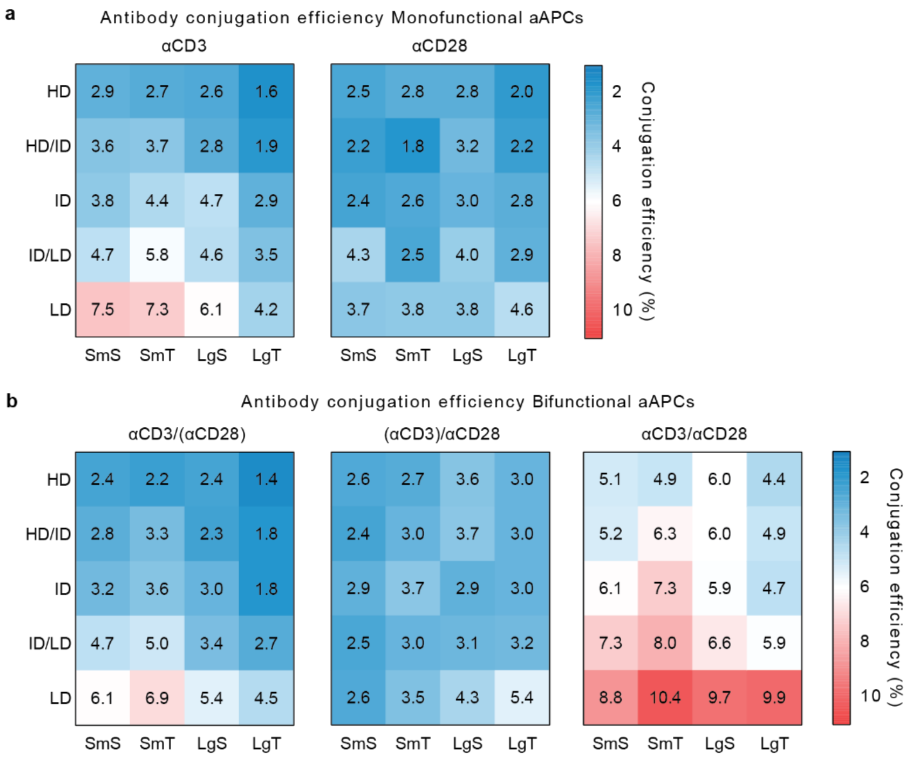

Figure S12. Antibody conjugation efficiencies (%) for aAPCs. Conjugation efficiency of αCD3 or αCD28 or their sum per polymersome morphology for (**a**) monofunctional aAPCs or (**b**) bifunctional aAPCs with different densities.

a Antibody Number Monofunctional aAPCs (Antibody number/particle)

αCD3

	SmS	SmT	LgS	LgT
HD	210	201	636	635
HD/ID	127	138	337	385
ID	68	82	286	290
ID/LD	42	54	138	177
LD	34	34	92	107

αCD28

	SmS	SmT	LgS	LgT
HD	359	417	1348	1594
HD/ID	157	134	771	912
ID	86	96	368	569
ID/LD	77	47	242	292
LD	33	35	116	236

b Antibody Number Bifunctional aAPCs (Antibody number/particle)

αCD3/(αCD28)

	SmS	SmT	LgS	LgT
HD	173	163	573	579
HD/ID	99	123	276	371
ID	57	67	183	181
ID/LD	42	47	105	138
LD	27	32	82	114

(αCD3)/αCD28

	SmS	SmT	LgS	LgT
HD	377	403	1752	2405
HD/ID	172	221	898	1232
ID	102	136	351	599
ID/LD	45	55	190	320
LD	23	32	130	273

αCD3/αCD28

	SmS	SmT	LgS	LgT
HD	550	566	2325	2983
HD/ID	271	344	1175	1602
ID	160	203	534	780
ID/LD	87	102	295	458
LD	51	64	212	387

Figure S13. Number of antibodies per particle for aAPCs. Number of αCD3 or αCD28 or their sum per polymersome morphology for (**a**) monofunctional aAPCs or (**b**) bifunctional aAPCs with different densities.

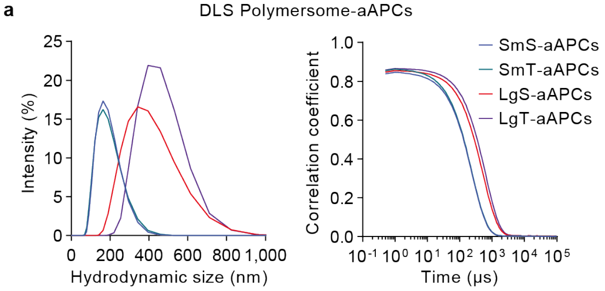

b

Polymersome-aAPCs characterization

Name	D_h (nm)	PDI	Particle concentration (particles/mL)	Surface area concentration (nm²/mL)
**SmS-aAPCs**	163	0.11	$4.0\cdot10^{11} \pm 4.4\cdot10^{10}$	$3.2\cdot10^{16} \pm 3.6\cdot10^{15}$
**SmT-aAPCs**	161	0.13	$3.9\cdot10^{11} \pm 4.8\cdot10^{10}$	$3.4\cdot10^{16} \pm 4.2\cdot10^{15}$
**LgS-aAPCs**	347	0.12	$1.2\cdot10^{11} \pm 1.5\cdot10^{10}$	$3.2\cdot10^{16} \pm 4.0\cdot10^{15}$
**LgT-aAPCs**	419	0.05	$1.1\cdot10^{11} \pm 1.3\cdot10^{10}$	$3.8\cdot10^{16} \pm 5.3\cdot10^{15}$

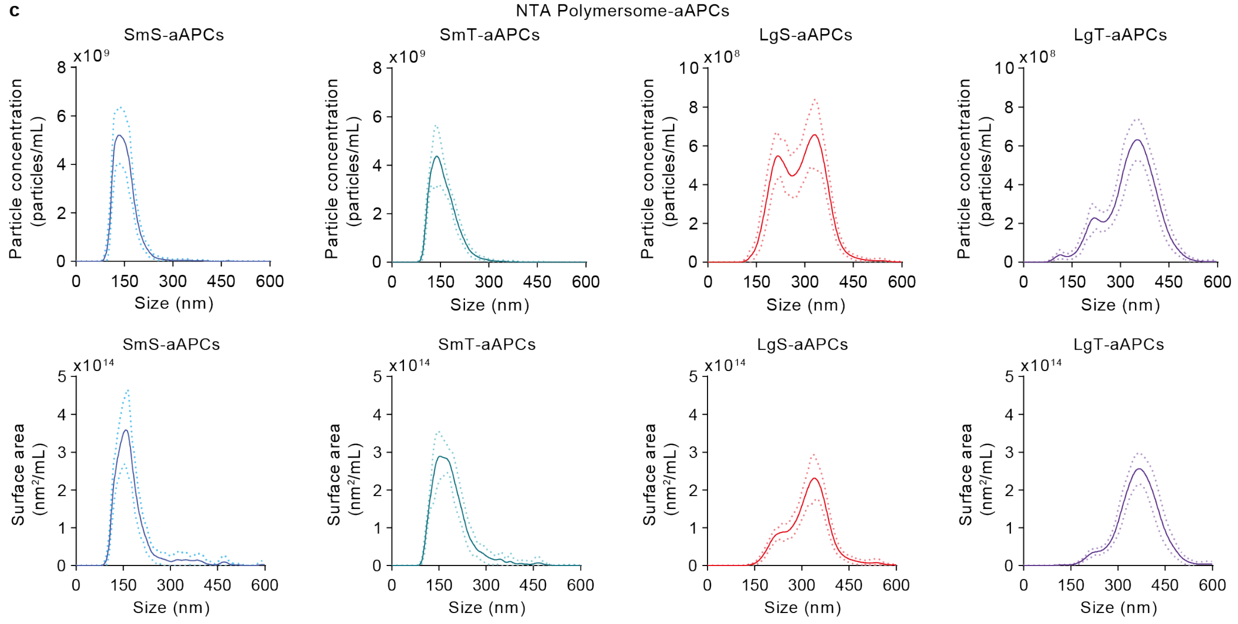

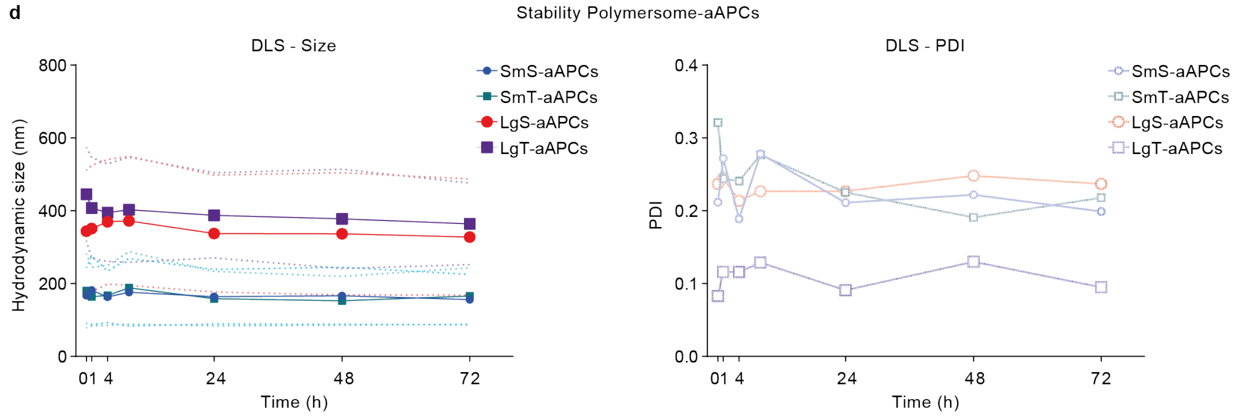

Figure S14. Analysis of aAPC integrity, particle characteristics and stability. **a**, DLS intensity profiles and correlograms of polymersome-aAPCs. **b**, Overview of characterization of polymersome-aAPCs, including hydrodynamic diameter (D_h), PDI and mean particle and surface area concentrations. **c**, Nanoparticle tracking analysis (NTA) characterization of aAPCs. Particle and surface area concentration as a function of particle size are displayed. **d**, DLS of aAPCs demonstrating their stability under physiological conditions (in X-VIVO™/1%HS at 37˚C). For both DLS and NTA in (**a-c**), the curves demonstrate the mean of the fifteen degrees of functionalization (both monofunctional and bifunctional) per polymersome morphology (n=15). The dotted line in (**c**) represents the SD. The dotted line in (**d**) represents the SD calculated using SD=√PDI·Size (n=3). Data represents mean ± SD.

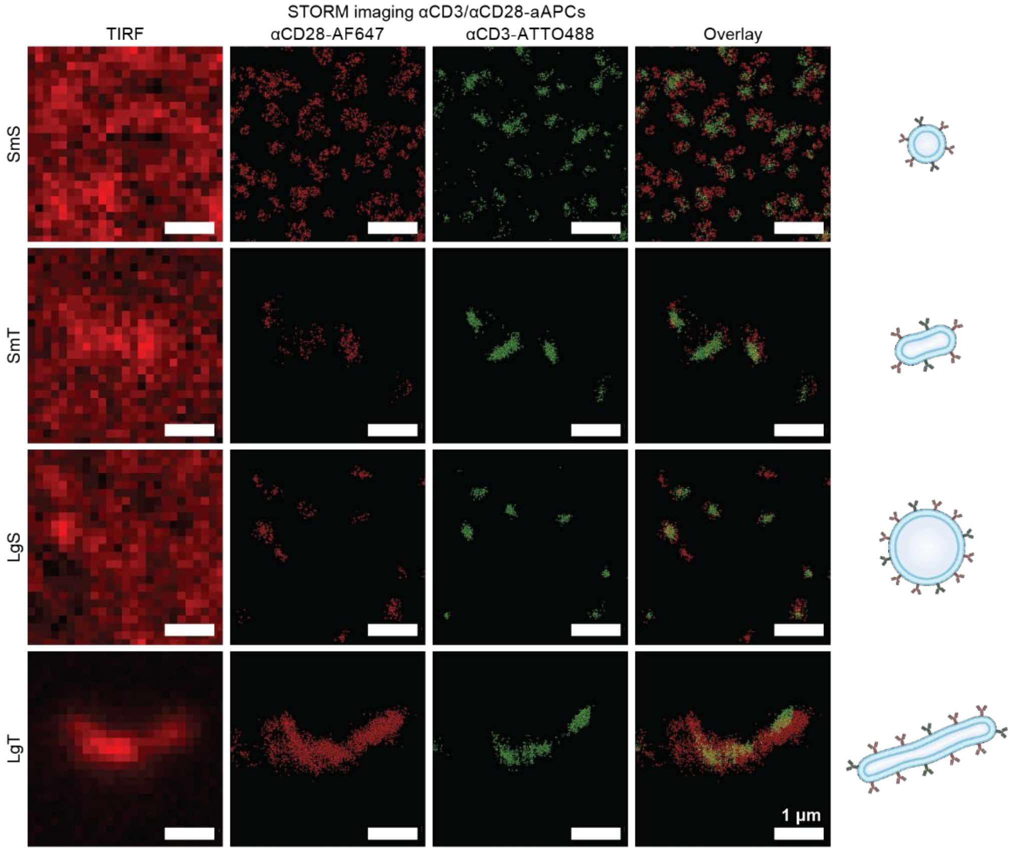

Figure S15. STORM imaging of bifunctional aAPCs indicates successful antibody conjugation on different polymersome morphologies. Scale bar = 1 µm.

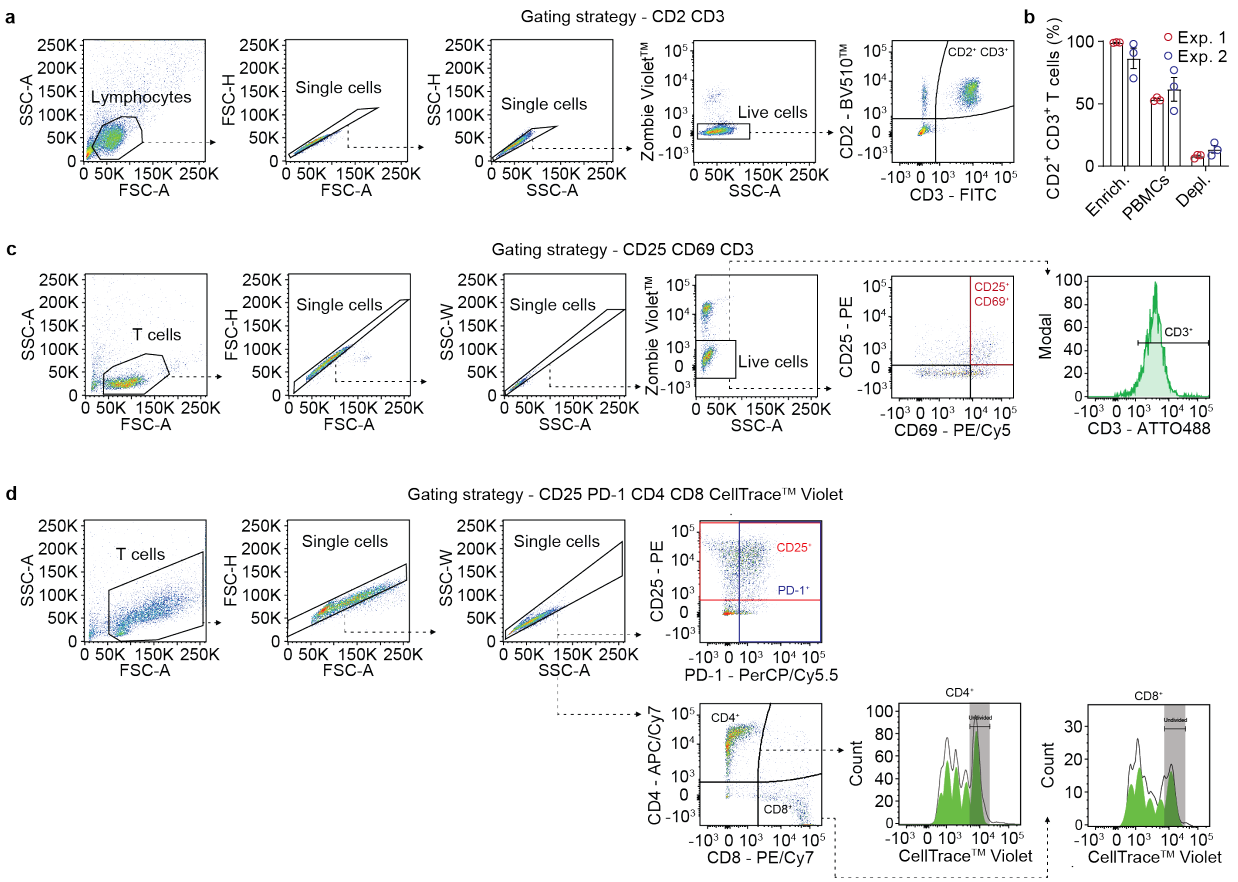

Figure S16. Flow cytometry gating strategies. **a**, Flow cytometry gating to determine the T cell population purity after Pan T cell isolation. Representative dot plots show gating on peripheral blood mononuclear cells (PBMCs) to exclude debris, single cells to exclude doublets, viable cells to exclude dead cells and $CD2^+$ $CD3^+$ cells to obtain the frequency of T cells in samples before (PBMCs) and after (enriched and depleted) isolation. **b**, Purity of T cell isolate. Data from three individual donors are presented as mean ± standard error (SE). **c**, Flow cytometry gating to quantify the co-expression of CD25 and CD69 and the binding of αCD3. Representative dot plots show gating on T cells, single cells, viable cells and $CD25^+$ $CD69^+$ cells to acquire the frequency of activated T cells after 24 hours of culture. Representative histogram shows fluorescence intensity of $\alpha CD3^+$ cells to determine the binding of the aAPCs after 6 hours of culture. **d**, Flow cytometry gating strategy to quantify the expression of CD25 and PD-1 and analyze proliferation of $CD4^+$ and $CD8^+$ T cell subsets through use of CTV™. Representative dot plots show gating on $CD25^+$ or PD-1^+ to determine T cell activation after 24 hours or 3 days, and gating on $CD4^+$ and $CD8^+$ subsets. CTV™ fluorescence histogram was analyzed to obtain the Division Index of $CD4^+$ or $CD8^+$ T cells after 3 days.

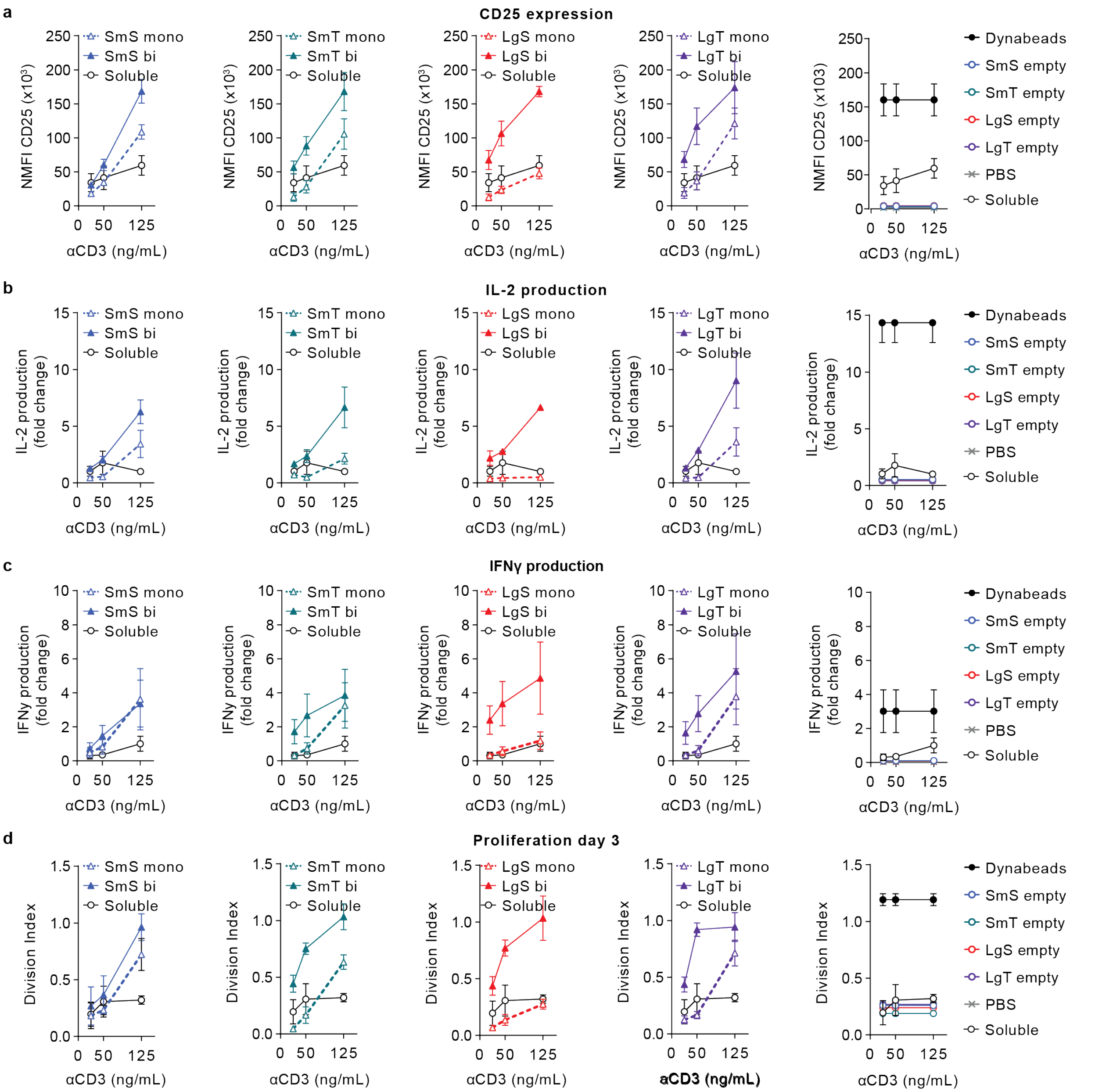

Figure S17. Bifunctional aAPCs enhance T cell activation compared to monofunctional aAPCs. T cells isolated from healthy donor buffy coats were stimulated with HD bifunctional (bi) or monofunctional (mono) aAPCs or soluble antibodies at 25, 50 and 125 ng/mL αCD3 concentrations and ~50, 100 and 250 ng/mL αCD28 concentrations. Unfunctionalized polymersomes (empty; 0 ng/mL αCD3) and Dynabeads™ (bead 1:1 cell) were used as negative and positive controls, respectively. **a**, Normalized CD25 expression (freq.·mean fluorescence intensity; NMFI), as determined with flow cytometry after 24 hours. **b**, **c**, Fold change in (**b**) IL-2 or (**c**) IFNγ production, relative to soluble αCD3+αCD28 at 125 ng/mL αCD3, as measured with ELISA after 24 hours. **d**, Division Index (average number of cell divisions) of CD4+ and CD8+ T cells (mean of subsets indicated) as determined through flow cytometric analysis of CTV™ fluorescence after three days of culture. All data is represented as mean ± SE (N=3 donors).

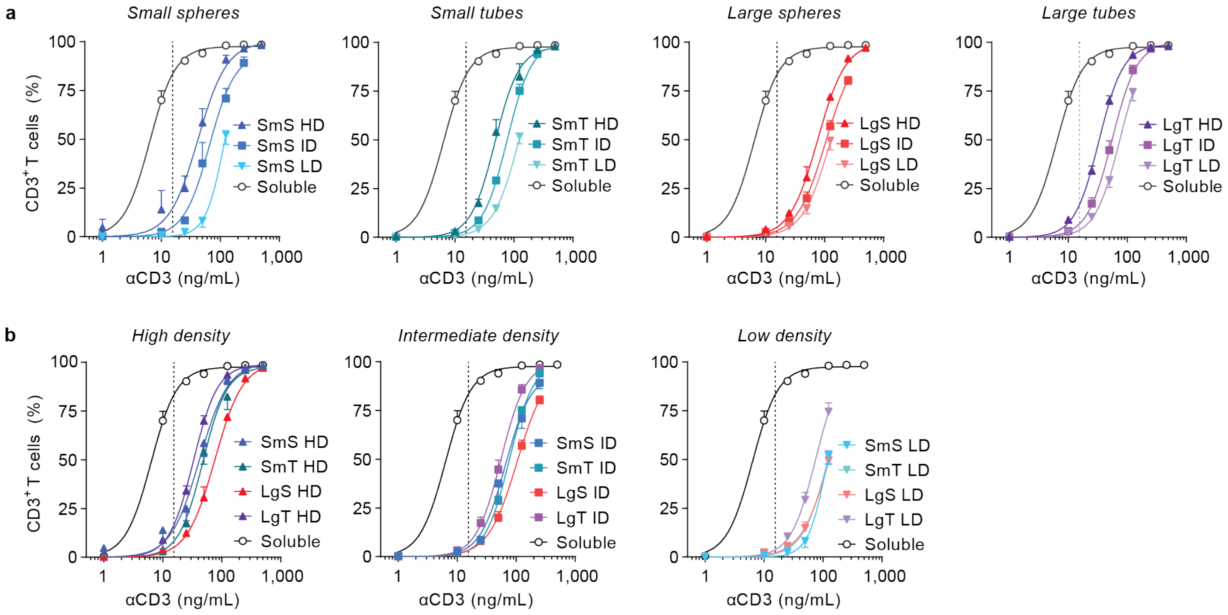

Figure S18. aAPC binding to T cells. T cells isolated from healthy donor buffy coats were stimulated with a library of aAPCs or soluble antibodies at a range of αCD3 (1-500 ng/mL) and αCD28 (~2-1,000 ng/mL) concentrations. Binding of aAPCs or soluble antibodies to T cells as determined with flow cytometry after 6 hours of culturing. **a**, Binding of aAPCs per polymersome morphology. **b**, Binding of aAPCs per density. Dotted line represents theoretical concentration at which cells should be saturated with αCD3 (~15 ng/mL, based on 124,000 CD3 molecules/T cell)[4]. All data is represented as mean ± SE (N=3 donors, n=2 replicates).

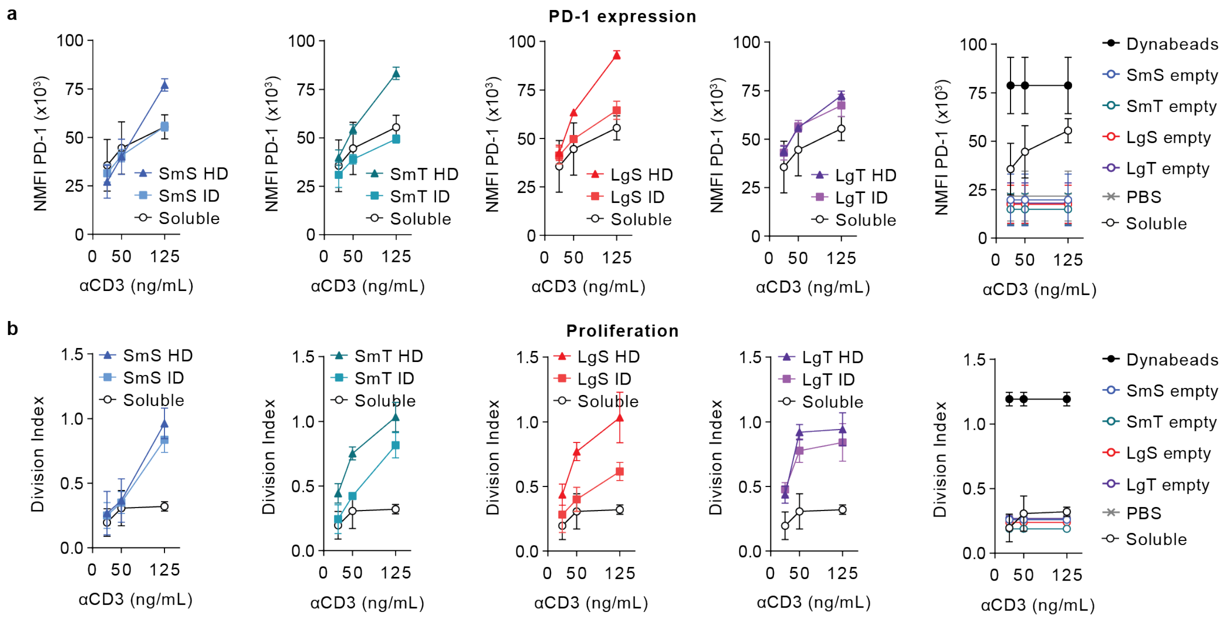

Figure S19. High ligand density enhances PD-1 expression and T cell proliferation after 3 days. T cells isolated from healthy donor buffy coats were stimulated for three days with a library of bifunctional aAPCs at high (HD) or intermediate (ID) density or soluble antibodies at 25, 50, and 125 ng/mL αCD3 concentrations, and ~50, 100, and 250 ng/mL αCD28 concentrations. Ligand densities and soluble antibody were compared for small spheres (SmS), large spheres (LgS), small tubes (SmT) and large spheres (LgT). Unfunctionalized polymersomes (empty; 0 ng/mL αCD3) and Dynabeads™ (bead 1:1 cell) were taken into account as negative and positive controls, respectively. **a**, Normalized PD-1 expression (freq.*mean fluorescence intensity; NMFI), as determined with flow cytometry after three days of culturing. HD enhances PD-1 expression compared to ID for SmS, SmT and LgS but not LgT. **b**, Division Index of $CD4^+$ and $CD8^+$ T cells (mean of subsets indicated) as determined through flow cytometric analysis of CTV™ fluorescence after three days of culture. HD enhances the proliferation compared to ID for SmT and LgS but not SmS and LgT. All data is represented as mean ± SE (N=3 donors).

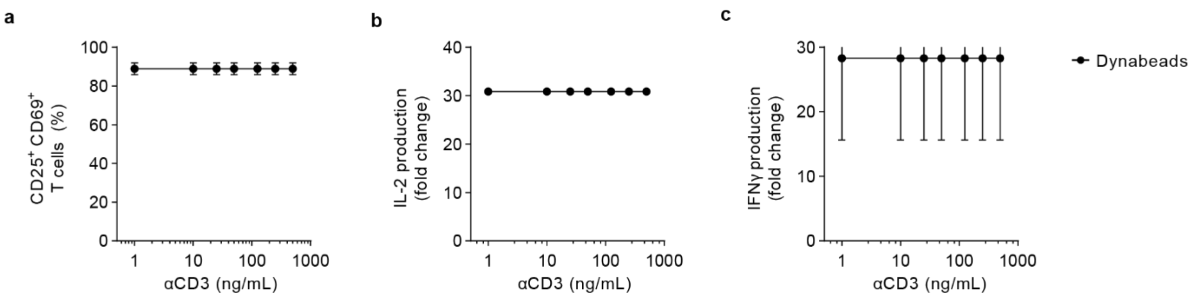

Figure S20. T cell activation by Dynabeads. T cells isolated from healthy donor buffy coats were stimulated with a library of bifunctional aAPCs with different polymersome morphologies or soluble antibodies at a range of αCD3 (1-500 ng/mL) and αCD28 (~2-1,000 ng/mL) concentrations (indicated in Figures 4 and 5). Dynabeads^{TM} (bead 1:1 cell) were taken into account as positive controls in T cell activation experiments. **a**, CD25 and CD69 co-expression (freq.) as determined by flow cytometry after 24 hours. **b**, **c**, Fold change in (**b**) IL-2 or (**c**) IFNγ production, relative to soluble αCD3+αCD28 at 125 ng/mL αCD3, as measured with ELISA after 24 hours. All data is represented as mean ± SE (N=3 donors, n=2 replicates).

REFERENCES

(1) Lohmeijer, B. G. G.; Pratt, R. C.; Leibfarth, F.; Logan, J. W.; Long, D. A.; Dove, A. P.; Nederberg, F.; Choi, J.; Wade, C.; Waymouth, R. M.; et al. Guanidine and Amidine Organocatalysts for Ring-Opening Polymerization of Cyclic Esters. *Macromolecules* **2006**, *39* (25), 8574–8583.

(2) Abdelmohsen, L. K. E. A.; Williams, D. S.; Pille, J.; Ozel, S. G.; Rikken, R. S. M.; Wilson, D. A.; van Hest, J. C. M. Formation of Well-Defined, Functional Nanotubes via Osmotically Induced Shape Transformation of Biodegradable Polymersomes. *J. Am. Chem. Soc.* **2016**, *138* (30), 9353–9356.

(3) Wauters, A. C.; Pijpers, I. A. B.; Mason, A. F.; Williams, D. S.; Tel, J.; Abdelmohsen, L. K. E. A.; Van Hest, J. C. M. Development of Morphologically Discrete PEG-PDLLA Nanotubes for Precision Nanomedicine. *Biomacromolecules* **2019**, *20* (1), 177–183.

(4) Ginaldi, L.; Matutes, E.; Farahat, N.; De Martinis, M.; Morilla, R.; Catovsky, D. Differential Expression of CD3 and CD7 in T-Cell Malignancies: A Quantitative Study by Flow Cytometry. *Br. J. Haematol.* **1996**, *93* (4), 921–927.
